# Competition-dominated microbial interactions and adaptive mutations enhance synthetic denitrifying community functions

**DOI:** 10.1128/mbio.02360-26

**Published:** 2026-10-21

**Authors:** Zhenrui Zhao, Ruiwen Hu, Min Zhou, Xiaotong Guan, Huaxia Huang, Lang Ding, Lu Qian, Yuzhen Ming, Mingyang Niu, Kun Wu, Xiaoli Yu, Qingyun Yan, Longfei Shu, Qiang He, Zhili He

**Affiliations:** 1 School of Marine Sciences, Marine Synthetic Ecology Research Center, Southern Marine Science and Engineering Guangdong Laboratory (Zhuhai), the China-ASEAN Belt and Road Joint Laboratory on Mariculture Technology, School of Environmental Science and Engineering, Sun Yat-sen University 590852, Zhuhai, China; 2 Environmental Genomics and Systems Biology Division, Lawrence Berkeley National Laboratory 1666, Berkeley, California, USA; 3 Department of Civil and Environmental Engineering, University of Tennessee 4292, Knoxville, Tennessee, USA; Georgia Institute of Technology, Atlanta, Georgia, USA

**Keywords:** synthetic denitrifying community, species richness, microbial interaction, adaptive mutations, community function, experimental evolution

## Abstract

Understanding how interspecific interactions shape ecosystem functioning is a fundamental and longstanding question in ecology, especially along an evolutionary trajectory. Here, we used experimental evolution to evaluate how microbial interactions and adaptive mutations shaped community-level functions in synthetic denitrifying communities (SDCs) along a species richness gradient (one, two, and six species). Our results showed that competitive microbial interactions were observed and significantly (*P* < 0.05) enhanced denitrification rates in the six-species SDCs during experimental evolution. Genome and metagenome re-sequencing analysis revealed the highest fixed non-synonymous and frameshift mutations within the six-species SDCs, among genes involved in nutrient transport, DNA repair, and secretion pathways. AlphaFold2 protein structure prediction, molecular docking, and Evo 2 host fitness prediction indicated that the fixed mutations could improve host strain fitness, and partial least squares path modeling (PLS-PM) further revealed that interspecific competition accelerated adaptive mutations, thereby reinforcing community-level functions, indicating potential Red Queen dynamics. This study offers novel insights into our understanding of eco-evolutionary mechanisms linking species richness, microbial interactions, adaptive mutations, and community functions, with important implications for active nitrogen removal and greenhouse gas mitigation in diverse ecosystems.

**IMPORTANCE:**

Microbial diversity is widely recognized as a key driver of ecosystem functioning, yet how species interactions shape ecosystem functions through evolutionary processes remains poorly understood. Using synthetic denitrifying communities with different levels of species richness, we show that interspecific competition can accelerate adaptive mutations and enhance community-level denitrification over evolutionary timescales. Communities with higher species richness accumulated more adaptive mutations, particularly in genes associated with nutrient transport, DNA repair, and secretion systems, ultimately improving microbial fitness and denitrification performance. Our findings provide direct experimental evidence that microbial competition can promote functional adaptation, consistent with the Red Queen hypothesis. This study advances our understanding of the eco-evolutionary mechanisms linking species richness, microbial interactions, adaptive evolution, and ecosystem functioning.

## INTRODUCTION

Ecosystem functioning emerges as a dynamic outcome of species diversity, interspecific interactions, and species-environment interplay, all of which are fundamentally shaped by evolutionary processes (1). Over the past few decades, evidence has accumulated for a positive relationship between biodiversity and ecosystem functioning across ecosystems (2–5). It was proposed that increasing species diversity can alter the probability of positive species interactions, resulting in disproportionately large changes in ecosystem functioning (3, 6, 7). Although these studies highlight the important role of high species richness and interspecific interactions in sustaining and promoting ecosystem functioning, current knowledge about how these ecological factors work together through evolutionary processes to affect ecosystem functioning over long-time scales remains limited.

Interspecific interactions play crucial roles in shaping community evolutionary trajectories over long timescales (8–11). For example, rapid prey evolution in response to oscillating predator density affected predator-prey (rotifer-algal) cycles in laboratory microcosms (12); plant-soil feedbacks mediated by mycorrhizal fungi influenced plant coexistence and evolutionary dynamics (13); virus-host coevolution led to single-nucleotide polymorphism (SNP) dynamics in viruses (14). As a well-known theory of coevolution, the Red Queen hypothesis describes that species must continually evolve new adaptations in response to evolutionary changes in other organisms to avoid extinction (15), highlighting interspecific interactions as persistent drivers of adaptive evolution. However, elucidating such molecular mechanisms remains challenging, largely due to the complexity of natural ecosystems and the difficulty in manipulating their evolutionary dynamics over extended timescales.

To address these challenges, synthetic microbial ecology has emerged as a powerful platform for us to dissect the evolutionary dynamics of synthetic microbial communities under controlled conditions (16, 17). As simplified analogs of natural ecosystems, these synthetic microbial communities could be constructed through bottom-up approaches and characterized by reduced complexity, high experimental controllability, and high reproducibility (18, 19). Especially, synthetic microbial communities provide a powerful tool for investigating how interspecific interactions shape ecosystem functioning through evolutionary processes due to their short generation times and large population sizes (20). For example, based on synthetic microbial communities comprising 1–12 species, researchers revealed that species interactions evolved to become less negative over time and resulted in enhanced community productivity, particularly in more diverse communities, highlighting the interactive effects of diversity and evolution on ecosystem functioning (21). Microbial species isolated from a beech tree hole and co-cultured in the laboratory for ~70 generations diverged in resource utilization, evolved to exploit each other’s metabolites, and consequently developed increasingly positive interspecific interactions that enhanced the overall community productivity (22). Recently, we also found that species richness was positively correlated with community-level functions using synthetic denitrifying communities (SDCs) (5) and that high phylogenetic diversity SDCs exhibited gradually enhanced functional stability during experimental evolution (23). However, how microbial interactions shape community functions and evolutionary dynamics remains largely unresolved.

Microbially driven denitrification is a crucial process in the global nitrogen (N) cycle, where nitrate (NO_3_⁻) is reduced to nitrite (NO_2_⁻), nitric oxide (NO), nitrous oxide (N_2_O), and ultimately to nitrogen gas (N_2_) through the activity of denitrifiers with functional genes *narG*, *nirS*/*nirK*, *norB,* and *nosZ*, respectively (24). This process not only plays an important role in N removal from eutrophicated ecosystems but also has great potential to mitigate N_2_O emissions (25, 26). Notably, denitrifiers differ in metabolic capacity, with some performing the complete denitrification process and others specializing in a subset of the denitrification metabolic pathway. Such metabolic partitioning necessitates interspecific interactions and renders denitrification a modular community-level process (27, 28). For example, a previous study showed that metabolic complementarity among denitrifiers enabled complete denitrification in soil and influenced N_2_O emissions, which depended on the functional compatibility among denitrifying strains (27). Therefore, denitrification efficiency may strongly depend on the diversity, composition, interaction, and evolutionary dynamics of denitrifying communities, making them an ideal model system for testing eco-evolutionary principles (29).

**Fig 1:**
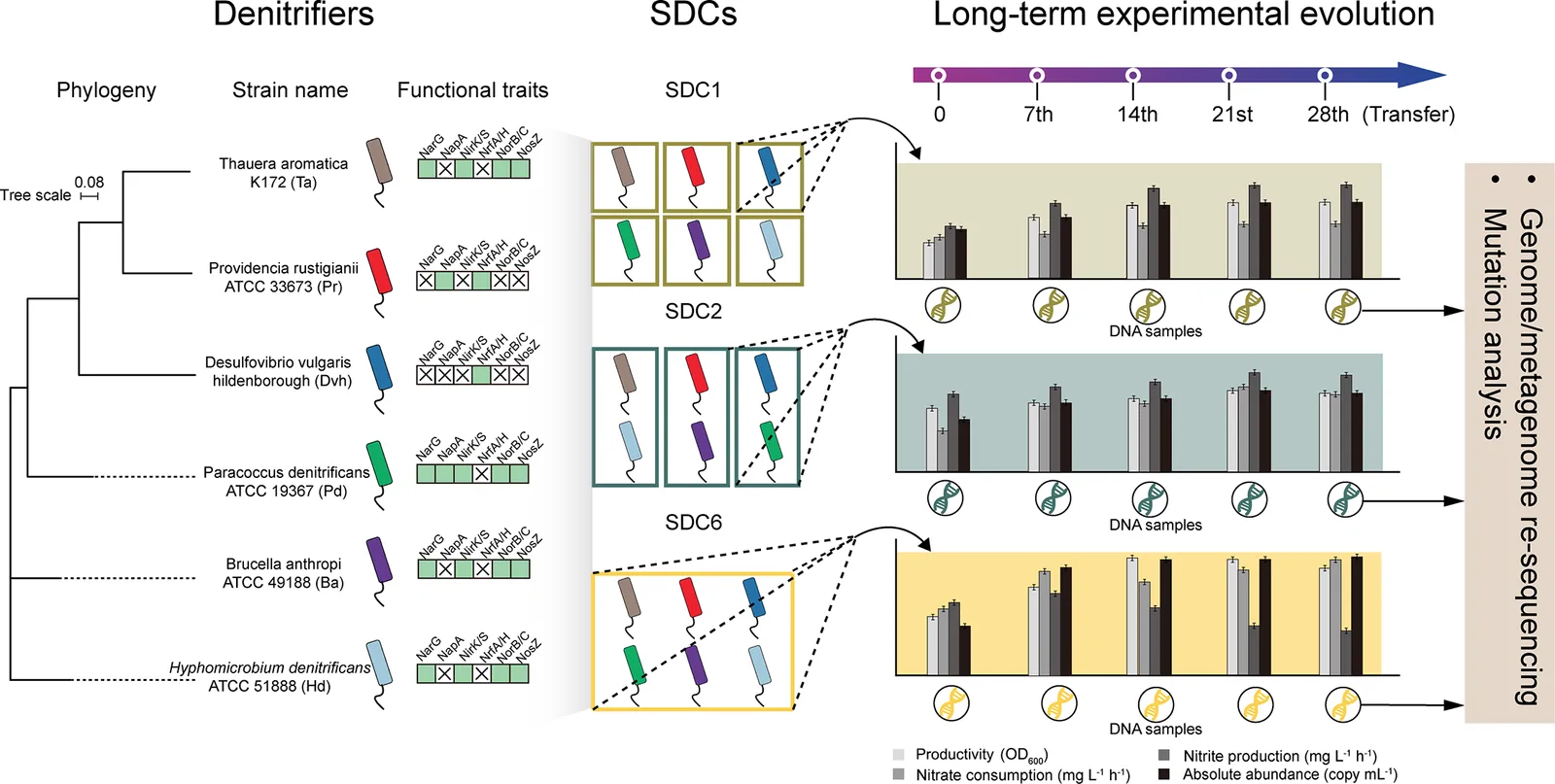
Workflow to explore how species richness, microbial interactions, and evolutionary dynamics jointly shape community functions in SDCs during long-term experimental evolution. Six bacterial strains with complementary nitrogen-transformation traits were used to construct the SDCs: *Thauera aromatica* K172 (Ta), *Providencia rustigianii* ATCC 33673 (Pr), *Desulfovibrio vulgaris* Hildenborough (DvH), *Paracoccus denitrificans* ATCC 19367 (Pd), *Brucella anthropi* ATCC 49188 (Ba), and *Hyphomicrobium denitrificans* ATCC 51888 (Hd). In the functional-trait matrix, green boxes indicate the presence of the corresponding gene, whereas crosses indicate its absence. NarG and NapA represent membrane-bound and periplasmic nitrate reductases, respectively; NirK and NirS represent copper-containing and cytochrome *cd*₁ nitrite reductases, respectively; NrfA/H represents a membrane-associated cytochrome *c* nitrite reductase complex involved in dissimilatory nitrite reduction to ammonium; NorB/C represents nitric oxide reductase; and NosZ represents nitrous oxide reductase. SDC1 consisted of six separate mono-cultures, with each strain cultivated independently. SDC2 consisted of three two-species communities: Ta–Hd, Pr–Ba, and DvH–Pd. SDC6 contained all six strains and was independently established in triplicate. The communities were serially transferred 28 times and sampled at transfers 0, 7, 14, 21, and 28 for measurements of productivity (maximum OD_600_), nitrate consumption, nitrite production, strain-specific abundance, and genome/metagenome resequencing. Strain-specific absolute abundance was quantified using quantitative PCR (qPCR).

In this study, we aimed to understand how species richness, microbial interactions, and evolutionary dynamics jointly shape community functions using synthetic microbial community and experimental evolution approaches. We hypothesized that high-richness synthetic denitrifying communities would enhance community functions over an evolutionary timescale as a consequence of accumulating beneficial mutations, especially in genes associated with nutrient uptake and core metabolisms driven by a Red Queen-like competition (14, 15, 21, 30, 31). To test these hypotheses, we constructed SDCs with different levels of species richness, comprising denitrifying bacterial taxa grown in a defined medium containing a mixture of carbon sources (Table S1) and tracked their community functions, microbial interactions, and adaptive evolutionary trajectories during a 6-month experimental evolution time (Fig. 1). Our results demonstrate that competitive microbial interactions and adaptive mutations enhanced community functioning over an evolutionary timescale, providing new strategies for active N removal and greenhouse gas mitigation in diverse ecosystems.

## RESULTS

### Productivity and denitrification rates of SDCs during the experimental evolution

**Fig 2:**
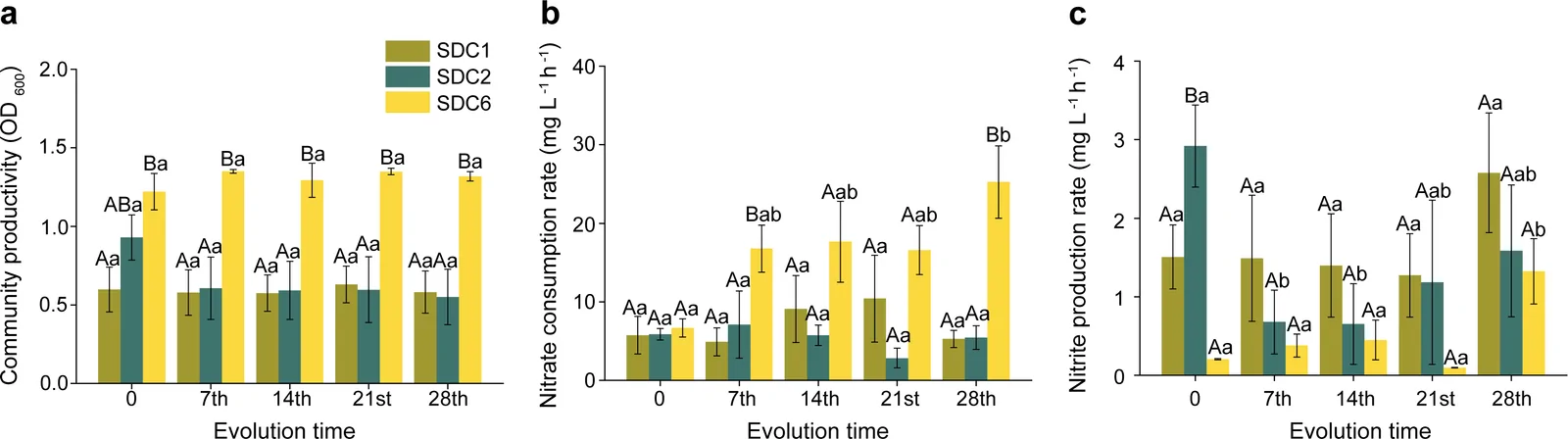
Changes in SDC productivity (**a**), nitrate consumption rate (**b**), and nitrite production rate (**c**) at different species richness levels during long-term experimental evolution. SDC1, SDC2, and SDC6 represent SDCs with richness levels of 1, 2, and 6, respectively. Different capital letters and lowercase letters represent significant differences in species richness and evolution time at the *P* < 0.05 level, respectively. Data are presented as mean ± standard error.

To assess functional dynamics during the experimental evolution, we measured community productivity, nitrate consumption rates, and nitrite production rates in all mono-cultures (SDC1: *Paracoccus denitrificans* [Pd], *Desulfovibrio vulgaris* Hildenborough [DvH], *Brucella anthropi* [Ba], *Providencia rustigianii* [Pr], *Hyphomicrobium denitrificans* [Hd], and *Thauera aromatica* K172 [Ta]), three two-species communities (SDC2: DvH–Pd, Pr–Ba, and Ta–Hd), and one six-species composition (SDC6: all six strains, with three biological replicates). A two-way analysis of variance (ANOVA) revealed that species richness had significant effects on community productivity (*F* = 32.507, *P* < 0.001), nitrate consumption rate (*F* = 11.318, *P* < 0.001), and nitrite production rate (*F* = 3.846, *P* = 0.029) (Fig. 2). At the 7th, 14th, 21st, and 28th transfer time points, the productivity was significantly (*P* < 0.05) higher in SDC6 than in SDC1 or SDC2 (Fig. 2a). Similarly, nitrate consumption rates were significantly (*P* < 0.05) elevated in SDC6 compared to SDC1 or SDC2 at the 7th and 28th transfers (Fig. 2b). The evolution time had no significant effects on productivity (*F* = 0.207, *P* > 0.05), nitrate consumption rates (*F* = 1.023, *P* > 0.05), or nitrite production rates (*F* = 1.358, *P* > 0.05) across all SDCs. However, nitrate consumption rates and nitrite production rates increased progressively over time, with rates at the 28th transfer significantly (*P* < 0.05) higher than those at the start time point (*T*_0_) in SDC6 (Fig. 2b and c). These results demonstrate that high-richness SDCs (e.g., SDC6) exhibited high productivity and gradually increased denitrification rates during the experimental evolution.

### Competitive interactions in SDC2 and SDC6

**Fig 3:**
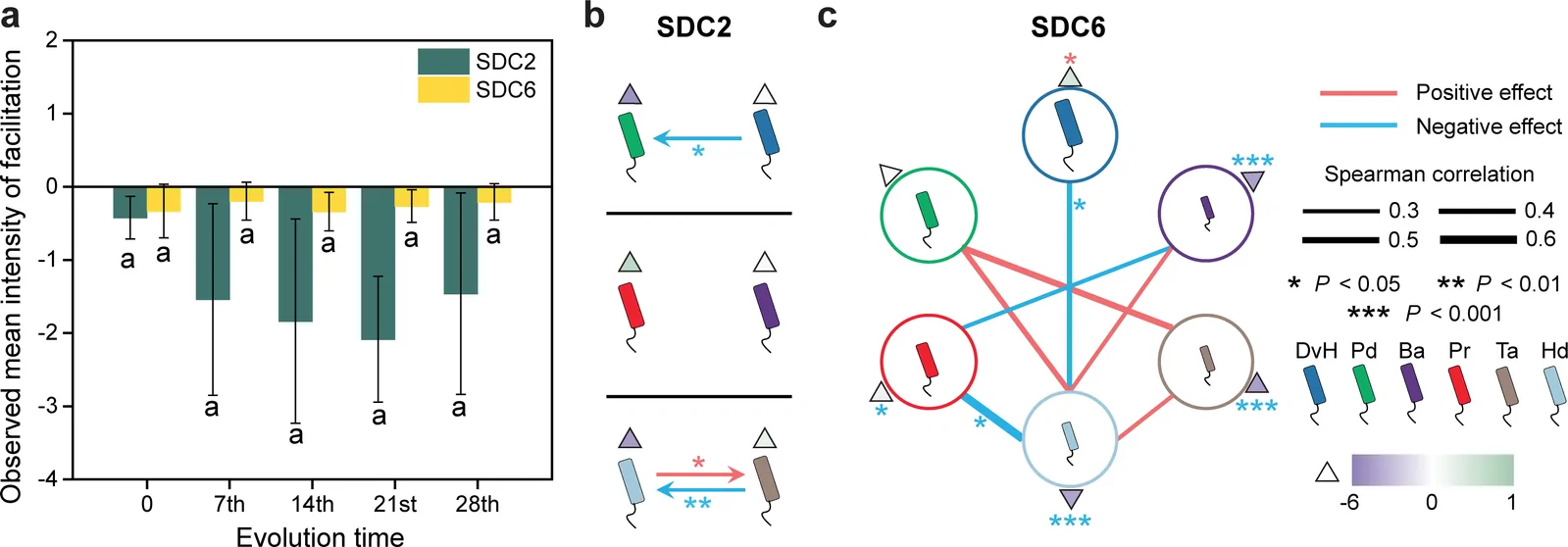
Microbial interactions in SDCs during experimental evolution. (**a**) The OIF in SDCs during experimental evolution. Communities were facilitative when OIF >0, antagonistic when OIF <0, and neutral if OIF = 0. Different lowercase letters represent significant differences in evolution time at the *P* < 0.05 level. Data are presented as mean ± standard error. Interaction between strains of the two-species (**b**) and six-species (**c**) SDCs during long-term experimental evolution. Red and blue lines represent positive and negative interactions, respectively. The thickness of the lines between strains in SDC6 represents the Spearman correlation coefficient. Triangles represent changes in absolute abundance of strains in co-cultures compared to mono-cultures. Triangle color is proportional to log_10_ (absolute abundance [co-culture]/absolute abundance [mono-culture]). *P < 0.05; **P < 0.01; ***P < 0.001.

To investigate microbial interactions during the experimental evolution, we quantified strain abundances in all SDCs using quantitative PCR (qPCR), calculated the observed mean intensity of facilitation (OIF) for SDC2 and SDC6, and constructed microbial interaction networks (Fig. S1; Fig. 3). In SDC2, OIF was −1.469, and evolution time had no significant (*F* = 0.316, *P* > 0.05) effects on OIF, indicating that competitive interactions predominated (Fig. 3a): DvH significantly (*P* < 0.05) suppressed Pd; Ta significantly (*P* < 0.01) inhibited Hd; Hd significantly (*P* < 0.05) promoted Ta; and Pr and Ba exhibited neutral interactions. The log_10_-transformed fold changes (relative to SDC1) for DvH, Pd, Pr, Ba, Hd, and Ta were −0.145, −5.094, 0.092, −4.631, 0.712, and 0.252, respectively (Fig. 3b). Competition was also evident in SDC6: OIF was −0.268, and evolution time had no significant (*F* = 0.060, *P* > 0.05) effects on OIF (Fig. 3a); the DvH abundance was significantly (*P* < 0.05) enhanced; the Ba, Pr, Hd, and Ta abundances were significantly (*P* < 0.05) reduced (Fig. 3c). The log_10_-transformed fold changes for DvH, Ba, Pr, Hd, Ta, and Pd were 0.445, −4.221, −4.401, −0.712, −4.445, and −0.198, respectively, and significant negative correlations were observed between Hd and DvH (*R* = −0.518, *P* < 0.05) and Hd and Pr (*R* = −0.625, *P* < 0.05) (Fig. 3c). These results indicate that microbial interactions in both SDC2 and SDC6 were dominated by competition, with a majority of strains being consistently suppressed over the evolutionary timescale.

### Mutation rates and evolutionary trajectories in all SDCs and individual populations

**Fig 4:**
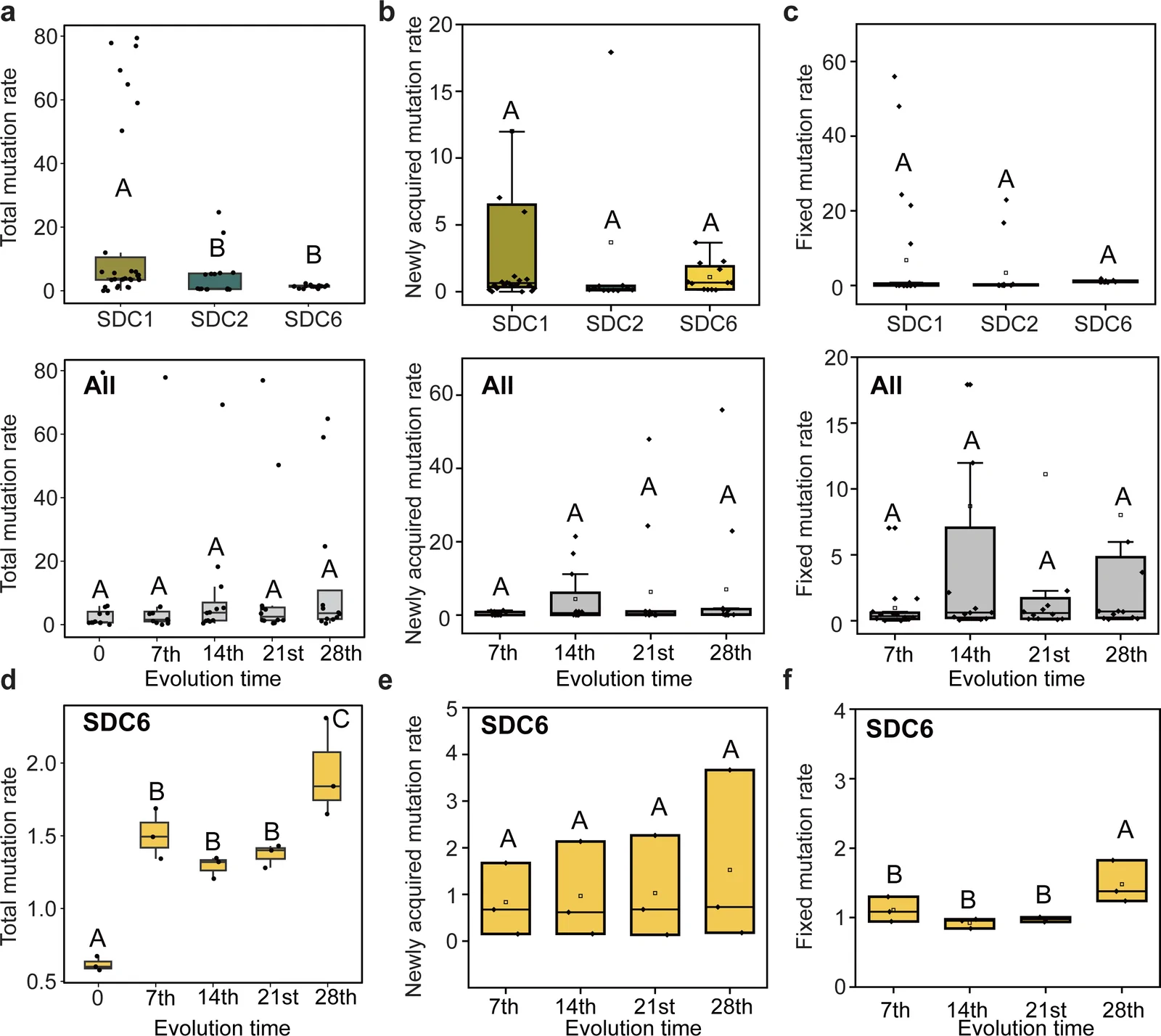
Effect of species richness and evolution time on changes in total (**a**), newly acquired (**b**), and fixed (**c**) mutation rates across all SDCs during the experimental evolution. Effect of evolution time on changes in total (**d**), newly acquired (**e**), and fixed (**f**) mutation rates in SDC6. Different capital letters represent significant differences in species richness or evolution time at the *P* < 0.05 level.

To understand mutations and evolutionary trajectories, we analyzed the total, newly acquired, and fixed mutations in all SDCs and individual populations (Tables S2 to S4). First, the total mutation rates were 18.70, 4.93, and 1.34 mutations per megabase (Mb) in SDC1, SDC2, and SDC6, respectively, being significantly (*P* < 0.05) higher in SDC1 than in SDC2 or SDC6 (Fig. 4a); the acquired mutation rates were 12.02, 3.70, and 1.09 mutations/Mb in SDC1, SDC2, and SDC6, respectively; and the fixed mutation rates were 6.80, 3.41, and 1.12 mutations/Mb in SDC1, SDC2, and SDC6, respectively. Second, at the individual population level, the total mutation rates of DvH, Pd, Ba, Pr, Ta, and Hd were 1.30, 3.90, 0.73, 6.58, 15.81, and 0.92 mutations/Mb, with the three highest mutation rates for Ta (73.66), Pr (24.25), and DvH (5.71) in SDC1 (Table S2; Fig. S2 and S5d); the newly acquired mutation rates of DvH, Pd, Ba, Pr, Ta, and Hd were 0.20, 0.35, 0.40, 8.17, 8.03, and 0.14 mutations/Mb, respectively, being highest in SDC1 for Ta (39.77), Pr (30.31), and Pd (0.72) (Fig. S3 and S5e); and the fixed mutation rates of DvH, Pd, Ba, Pr, Ta, and Hd were 0.01, 0.05, 0.51, 7.81, 2.36, and 0.14 mutations/Mb across all SDCs, respectively, with the highest mutation rates for Pr (28.77), Ta (11.70), and Pd (0.14) in SDC1 (Fig. S4 and S5f). One-way ANOVA analysis showed that the total and newly acquired mutation rates of strains Pr and Ta were significantly (*P* < 0.05) higher than those of the other four strains, with Pr also displaying the highest fixed mutation rate (Fig. S5a through c). Furthermore, two-way ANOVA analysis across all richness treatments revealed that species richness had a significant (*F* = 3.591, *P* = 0.036) effect on the total mutation rate, but evolution time had no significant (*P* > 0.05) effect on the total mutation rate; neither species richness nor evolution time had significant (*P* > 0.05) effects on the newly acquired or fixed mutation rates (Table 1). A separate one-way ANOVA within SDC6 showed that evolution time had a significant (*P* < 0.05) effect on total mutation rates and fixed mutation rates, but not on newly acquired mutation rates. A significant (*P* < 0.05) increase at the 28th transfer compared to earlier time points was also observed for total mutation rates and fixed mutation rates (Fig. 4f), revealing that total mutation rates and fixed mutation rates increased progressively over evolution time in SDC6. Additionally, to further elucidate the relationships between total, newly acquired, and fixed mutation rates with community functions, we performed Spearman correlation analysis, showing that newly acquired and fixed mutation rates were significantly (*P* < 0.05) positively correlated with productivity and nitrate consumption rates (Fig. S6).

**TABLE 1:** Two-way ANOVA showing the effect of species richness and evolution time on changes in total, newly acquired, and fixed mutation rates in different SDCs during the experimental evolution.

Parameter	Total mutation rate		Newly acquired mutation rate		Fixed mutation rate	
	*F*	*P*	*F*	*P*	*F*	*P*
Richness	3.591	**0.036^*a*^**	1.618	0.212	0.902	0.415
Evolution time	0.107	0.980	0.310	0.818	0.405	0.750
Richness × evolution time	0.070	1.000	0.338	0.912	0.328	0.918

^*a*^ Bold values indicate statistically significant effects (*P* < 0.05).

### The dynamics of fixed mutations

**Fig 5:**
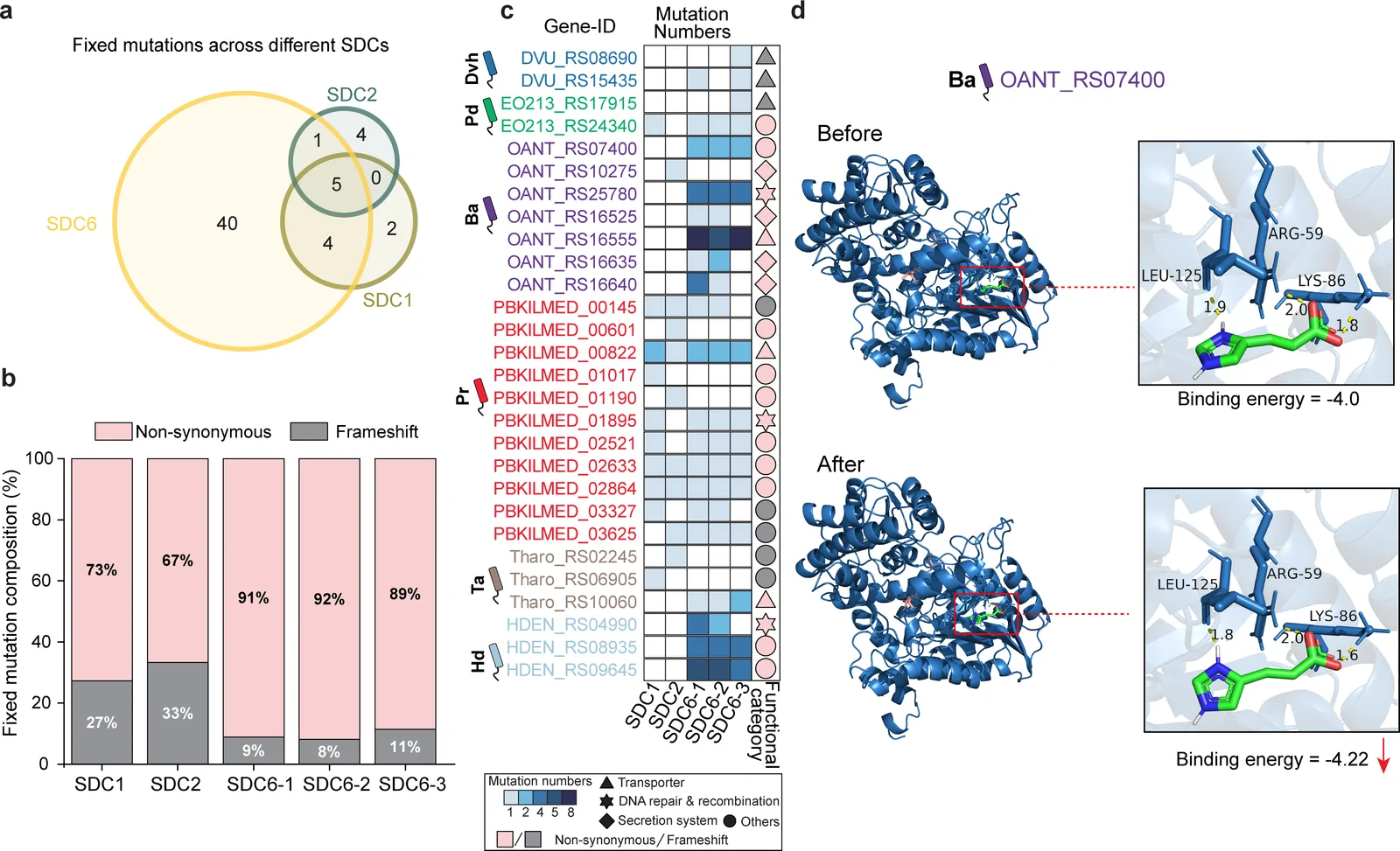
Fixed non-synonymous and frameshift mutations with known functions in different SDCs during the experimental evolution. Venn diagram (**a**) shows mutations that were common and unique to different SDCs. Column diagram (**b**) shows non-synonymous (red) and frameshift (gray) mutations in different SDCs. Heatmap (**c**) shows mutations in six strains in different SDCs. Different functional categories are represented by different shapes, with triangles representing transporters, six-pointed stars representing DNA repair and recombinant proteins, diamonds representing secretion system proteins, and circles representing other functional categories. Protein structure prediction and comparison of urocanate-binding energies (kcal/mol) before and after mutation in OANT_RS07400 (urocanate hydratase HutU) based on molecular docking analysis (**d**).

We further analyzed the temporal dynamics of fixed non-synonymous and frameshift mutations detected at the last two transfers and identified a total of 56 fixed mutations mapped to 28 genes (Table 2) with known functions: 2 were unique to SDC1, 4 were unique to SDC2, and 40 were unique to SDC6 (Fig. 5a). Non-synonymous mutations were more frequent than frameshift mutations across all SDCs (Fig. 5b). The fixed mutations were highly enriched in genes for nutrient transport (15 mutations), DNA repair and recombination (10), and secretion systems (8) in SDC6 (Fig. 5c). At the individual population level, a total of 2, 5, 6, 14, 22, and 54 fixed non-synonymous and frameshift mutations were identified in Pd, DvH, Ta, Ba, Hd, and Pr, respectively (Table S4). For example, a frameshift mutation in the DVU_RS15435 of DvH encoding an oligopeptide/dipeptide ABC transporter ATP-binding protein was fixed in SDC6-1 and SDC6-3, which remained consistently above 0.9 frequency across 14th, 21st, and 28th transfers (Fig. S7). In Pd, a frameshift mutation in EO213_RS17915 (iron chelate uptake ABC transporter family permease subunit) was fixed in SDC6-3, with the allele frequency (AF) exceeding 0.9 at both 21st and 28th transfers (Fig. S7). Non-synonymous mutations were fixed in several genes in Ba, such as OANT_RS07400 (*hutU*, urocanate hydratase, fixed in all SDC6 replicates, Fig. S7), OANT_RS16525 (*traG*, conjugal transfer protein), OANT_RS16555 (*arsB*, arsenite efflux transporter), OANT_RS16635 (*trbB*), and OANT_RS16640 (*trbE*). In Ta, a mutation in Tharo_RS10060 (*actP*, cation acetate symporter) was fixed in SDC6-1 and SDC6-3 with an allele frequency of 1.0 at both the 21st and 28th transfers (Fig. S7). In Hd, multiple mutations associated with transcriptional regulation and protein quality control were fixed in the genes: *rpoC* and *rpoD* (RNA polymerase subunits) and *clpX* (ATP-dependent Clp protease), all of which were present in at least two SDC6 replicates. Notably, the allele frequencies of five *clpX* mutation sites exceeded 0.98 at every transfer point (Fig. S7). These findings indicate that high-richness SDCs not only harbored the greatest number of functionally relevant fixed mutations but also exhibited recurrent fixation of identical mutations across replicates, suggesting strong parallel evolutionary trajectories and convergent functional adaptation.

**TABLE 2:** Functionally annotated fixed frameshift or non-synonymous mutations of six strains during the experimental evolution

Strain	Gene ID	Predicted function
DvH	DVU_RS08690	Hemolysin family protein
	DVU_RS15435	Oligopeptide/dipeptide ABC transporter ATP-binding protein
Pd	EO213_RS17915	Iron chelate uptake ABC transporter family permease subunit
	EO213_RS24340	AAA family ATPase
Ba	OANT_RS07400	Urocanate hydratase HutU
	OANT_RS10275	Preprotein translocase subunit SecY
	OANT_RS25780	Recombinase family protein
	OANT_RS16525	Conjugal transfer protein TraG
	OANT_RS16555	ACR3 family arsenite efflux transporter ArsB
	OANT_RS16635	Conjugal transfer protein TrbB
	OANT_RS16640	Conjugal transfer protein TrbE
Pr	PBKILMED_00145	Aspartate racemase
	PBKILMED_00601	D-malate dehydrogenase (decarboxylating) DmlA
	PBKILMED_00822	P-hydroxybenzoic acid efflux pump subunit AaeB
	PBKILMED_01017	Pyridoxal phosphate phosphatase YbhA
	PBKILMED_01190	Leucine-responsive regulatory protein Irp
	PBKILMED_01895	DNA-binding protein H-NS Hns
	PBKILMED_02521	Fimbria adhesin protein MrkD
	PBKILMED_02633	Cell division protein FtsA
	PBKILMED_02864	Sulfite reductase (NADPH) flavoprotein alpha-component CysJ
	PBKILMED_03327	Lipoprotein NlpI
	PBKILMED_03625	GalNAc-α-(1→4)-GalNAc-α-(1→3)-diNAcBac-PP-undecaprenol α-1,4-N-acetyl-D-galactosaminyltransferase PglH
Ta	Tharo_RS02245	DEAD/DEAH box helicase
	Tharo_RS06905	Putative Fe-S cluster assembly protein SufT
	Tharo_RS10060	Cation acetate symporter ActP
Hd	HDEN_RS04990	DNA-directed RNA polymerase subunit beta RpoC
	HDEN_RS08935	RNA polymerase sigma factor RpoD
	HDEN_RS09645	ATP-dependent Clp protease ATP-binding subunit ClpX

### Impacts of fixed mutations on gene functions

To evaluate the impact of these fixed mutations on gene functions, we predicted protein structures using AlphaFold2 and compared their pre- and post-mutation structures (Fig. S8). The most pronounced structural changes were observed in the proteins OANT_RS25780 (recombinase family protein, RMSD = 30.610), PBKILMED_00145 (aspartate racemase, 8.016), PBKILMED_03327 (lipoprotein NlpI, 2.599), Tharo_RS06905 (Fe-S cluster assembly protein SufT, 1.261), and DVU_RS08690 (hemolysin family protein, 1.239). To reveal the fitness effect of fixed mutations on microbial strains, Evo 2 analysis showed the five highest predicted effects on host fitness: Tharo_RS06905 (impact score = 3.50), PBKILMED_01190 (leucine-responsive regulatory protein lrp, 2.60), PBKILMED_03327 (1.38), DVU_RS15435 (1.35), and DVU_RS08690 (1.09) (Table S5). To further understand the functional impact of fixed mutations on ligand binding, molecular docking analysis was performed. The results revealed that the mutation in *hutU* gene could result in a lower binding energy for the urocanate ligand (from −4.0 to −4.22 kcal/mol), corresponding to an approximate 5.5% increase in binding strength (Fig. 5d). Similarly, the mutation in DVU_RS15435 could lead to a reduced binding energy for the ATP ligand (from −1.32 to −1.46 kcal/mol), representing an approximate 10.6% increase in binding affinity (Fig. S9). The results suggest that these mutations could enhance the interaction between the proteins and their respective ligands, potentially improving their functional activity.

### Competition-dominated mutations shape community functions

**Fig 6:**
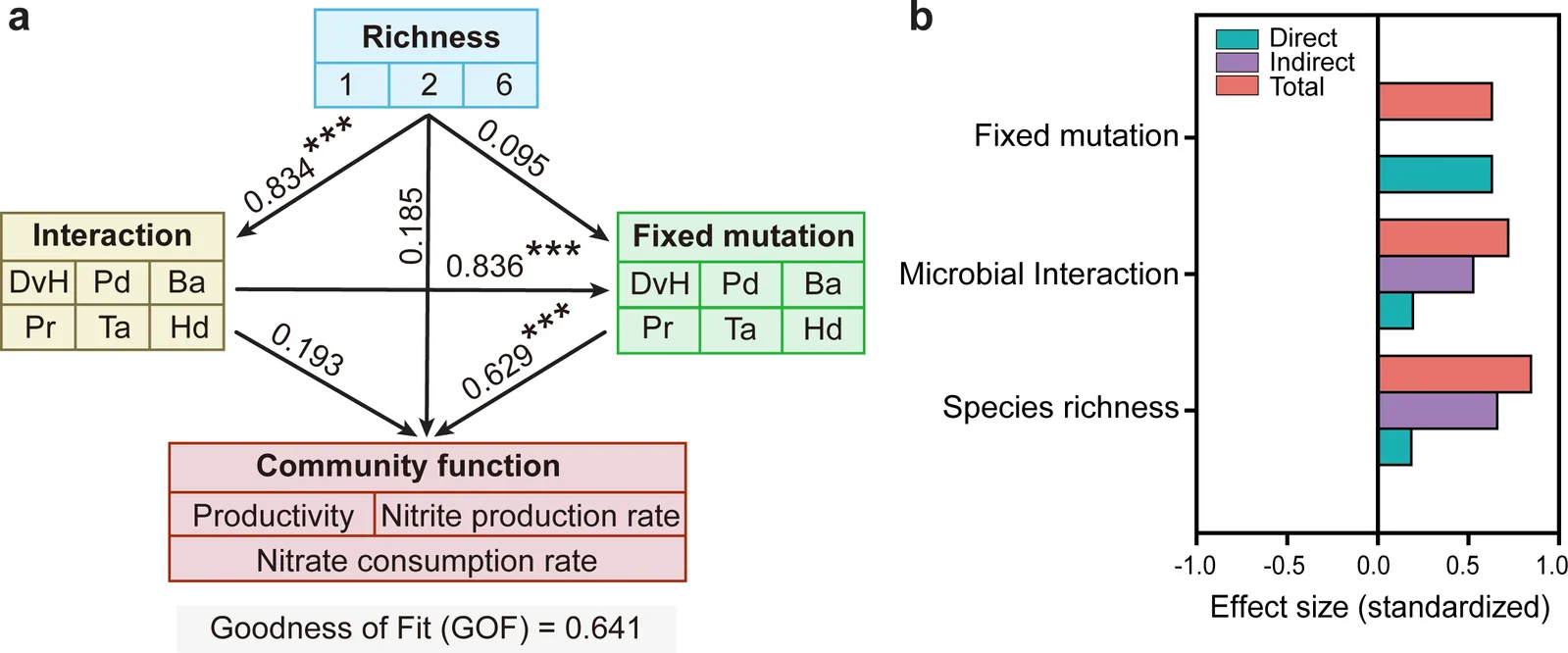
Eco-evolutionary mechanisms linking species richness to community functioning. (**a**) The PLS-PM analysis showing the relationships between species richness, microbial interaction, fixed mutation, and community function in SDCs during the evolution process. (**b**) The effect size of species richness, microbial interaction, and fixed mutation on community functioning. DvH, *Desulfovibrio vulgaris* Hildenborough; Pd, *Paracoccus denitrificans*; Ba, *Brucella anthropi*; Pr, *Providencia rustigianii*; Ta, *Thauera aromatica* K172; Hd: *Hyphomicrobium denitrificans*. ***P < 0.001.

To uncover the causal relationships among species richness, microbial interactions, fixed mutations, and community function during the experimental evolution, we employed partial least squares path modeling (PLS-PM; Fig. 6). Across all SDCs, species richness had a strong positive influence on microbial interactions (direct effect size = 0.834; *P* < 0.001) (Fig. 6a), indicating that increased richness intensified interactions dominated by interspecific competition. However, species richness had no significant direct effects on fixed mutation (0.095; *P* = 0.547) or community function (0.185; *P* = 0.084) (Fig. 6a), although it exerted a strong indirect positive effect on community function (0.659) mediated through microbial interaction and fixed mutation (Fig. 6b). Notably, microbial interaction had a significant (0.836; *P* < 0.001) positive effect on fixed mutation, and fixed mutation emerged as the dominant predictor of community function with a significant (0.629; *P* < 0.001) positive effect size (Fig. 6a), suggesting that the functional performance of microbial communities was primarily driven by evolutionary changes shaped by interspecific interactions, particularly competition-dominated mutations in this case.

## DISCUSSION

Understanding how species richness, microbial interaction, and evolutionary process jointly regulate ecosystem functioning is a central scientific challenge in ecology. While diverse communities are widely recognized to exhibit higher productivity and stability, the eco-evolutionary mechanisms underlying these patterns remain unclear. Here, we demonstrated that high-richness SDCs not only influenced ecological processes but also shaped evolutionary trajectories driven by microbial competition and mutation accumulation, thus enhancing community functions. During the experimental evolution, competitive interactions were dominant in high-richness SDCs, with a gradually increasing rate of fixed mutations, which was positively correlated with enhanced nitrate consumption. Together, these results support our core hypothesis that high-richness SDCs could enhance community functions during an experimental evolution through the accumulation of beneficial mutations driven by a Red Queen-like competition.

The positive relationship between species richness and ecosystem functioning has been extensively studied in macroecological systems (3, 32, 33). In most cases, high-richness communities are able to promote productivity, stability, and resource use efficiency, but the underlying mechanism and long-term persistence of these effects remain debatable (34). For example, high plant diversity strengthened rhizosphere microbial interactions and carbon use efficiency in agricultural soils (32); biodiversity across trophic levels improved productivity and stability in grasslands and forests (3, 33); long-term grassland experiments showed that diversity-driven biomass gains accumulated over time (35). By contrast, relatively few studies have investigated these patterns in microbial systems (2, 36–40). For example, soil biodiversity (bacteria, fungi, protists, and invertebrates) was found to be positively correlated with multiple ecosystem functions (2, 36–38); leaf bacterial diversity mediated plant diversity-ecosystem function relationships (41); the reduced diversity of denitrifying communities limited functional redundancy and compromised denitrification functions (39, 40). In this study, we found that high-richness SDCs exhibited consistently high productivity and gradually increasing denitrification rates, which is consistent with previous biodiversity–ecosystem functioning studies in both plant (7, 35, 42–49) and microbial communities (21, 22, 50–52). This outcome likely reflects the combined effects of ecological complementarity and evolutionary adaptation. Ecologically, high-richness communities display resource-use complementarity, functional redundancy, and complex interspecies interactions that enable different strains to exploit diverse substrates or occupy partially overlapping niches (53–55), thereby enhancing the community-level productivity. More importantly, our study expands classical biodiversity–ecosystem functioning relationship into an evolutionary trajectory, indicating that high diversity microbial communities are more likely to enhance ecosystem functioning over a long timescale. However, as high-richness communities inherently generate more complex interspecies interactions, elucidating how they enhance community functions along evolutionary trajectories is critical.

The functioning of microbial communities is shaped not only by species richness and composition but also by their ecological interactions within a community (56), including facilitation and competition (57). Previous studies showed that microbial competition is common (11), while facilitation tends to emerge under harsh conditions to promote coexistence (58). For example, most interspecies interactions were competitive in 180 two-species communities studied (59); synthetic microbial communities of 1–12 species revealed that species interactions became less negative over time, leading to increased community productivity (21); competition selected for strains with improved metabolic traits or niche partitioning during a long-term microbial evolution (22, 60, 61). In this study, we found that microbial interactions were predominantly competitive in the co-cultures, and underlying mechanisms were potentially compatible with the Red Queen hypothesis (11, 62–67). The overlap of electron acceptor utilization among community members could intensify selection pressure, forcing community members into an evolutionary “arms race,” in which continuous adaptation was required to maintain their fitness. More importantly, this study provides evidence that microbial interactions (e.g., competition) occurred at the population level, enabling individual populations to persist and co-evolve under controlled experimental conditions; thus, the community-level function (e.g., nitrate utilization) was improved. These population- and/or strain-level interactions highlight how the interplay between ecological competition and evolutionary adaptation jointly shapes the community functioning, which is not explained by species richness alone. Further understanding such underlying mechanisms at the community, individual population, and gene levels is important.

Adaptive mutations are fundamental drivers of microbial adaptation and evolution toward community function improvements (68, 69). In most cases, the functional improvement of microbial communities is linked to the accumulation of beneficial mutations that alter microbial composition, metabolism, stress responses, and ecological interactions (70–74). First, at the community level, the drift-barrier hypothesis posits that natural selection tends to refine trait performance up to a barrier beyond which further beneficial mutations are unlikely to overcome genetic drift, favoring the evolution of low mutation rates, thus sustaining the community and ecosystem stability (75, 76). In multi-species communities, microbial interaction, functional redundancy, and metabolic complementarity reduced selection pressure on community members, while in mono-cultures, a single species requires rapid mutations to adapt to the environment due to the lack of competitors or complementary species (30, 77). In this study, we found that the total mutation rate was lower in high-richness SDCs, and more interestingly, high-richness SDCs displayed a gradual increase in fixed mutation rates, which was positively correlated with enhanced nitrate consumption. These findings provide a novel perspective by directly linking species richness to the community-level mutation rate and functional stability, and such an eco-evolutionary relationship has rarely been explored before. Therefore, our results reinforce the notion that microbial interactions constrain the community evolutionary rate, selectively increase adaptive mutations, and ultimately shape community functioning.

Second, microbial interactions can also modulate evolutionary dynamics for individual populations (58, 74, 77–81). For example, a previous study has shown that the ecological stability of a co-culture community of *Saccharomyces cerevisiae* and *Lactobacillus plantarum* was maintained by constraining the adaptive trajectories of individual species (77); a mutualistic co-culture of *Desulfovibrio vulgaris* and *Methanococcus maripaludis* improved the community productivity and altered the evolution of *M. maripaludis* after 300 generations (79); in the resource-competition co-culture of *Escherichia coli* and *Saccharomyces cerevisiae*, competitive interaction promoted adaptive mutations, which ultimately facilitated their stable coexistence after ~1,000 generations of coevolution (80). In this study, the total mutation rate of six strains was higher in the mono-cultures than in their mixed cultures, while the rates of newly acquired mutations and fixed mutations varied among different populations. Importantly, our study demonstrates that species interactions can slow the evolutionary rate at both the population and community levels, revealing the community stabilizing role of biological interactions.

Third, microbial interactions could direct adaptive mutations to functionally relevant genomic regions or genes (82, 83). For example, in the study of diatom-*Richelia* symbioses, microbial interactions drove the specific loss of nitrate assimilation pathways (*narB*, *nirA*) and α-tocopherol synthesis pathways in endosymbionts but preserved the nitrogenase gene cluster (*nif*), thereby optimizing metabolic efficiency and enhancing host dependence (82); the mutations of *fhuA* and *btuB* genes in *E. coli* altered co-culture dynamics with *S. cerevisiae* (80); when coculturing *Staphylococcus xylosus* and *Debaryomyces hansenii* for 450 generations, the mutations in *SigB*, *Agr*, and *WalRK* operons were identified in *S. xylosus*, thus altering pigment and biofilm production, spreading, stress tolerance, and metabolism (83). In this study, high-richness SDCs harbored the highest number of fixed mutations, which were highly enriched in the genes for nutrient transport, DNA repair and recombination, and secretion systems. For example, the DvH population accumulated mutations in ABC transporters for oligopeptide uptake, potentially enhancing substrate affinity and host fitness, and Pd acquired mutations in iron-chelating transporters. These two changes could confer competitive advantages in nutrient acquisition or enhance antagonistic capabilities in resource-limited environments (84–86). In Ba, we also detected mutations in *hutU* (urocanase), which could strengthen urocanate binding, potentially broadening N scavenging from alternative substrates. Previous studies showed that the mutations of *virB* operon genes *traG*, *trbB*, and *trbE* encoding the components of a type IV secretion system (T4SS) could adopt virulence-related persistence strategies under competitive stress conditions (87), while the mutations in the *arsB* gene, typically linked to arsenic resistance, were detected despite no arsenic in the growth environment, indicating potential alternative roles in heavy metal detoxification or membrane transport (88). Additionally, the non-synonymous mutations of Ta were detected in the *actP* gene encoding a cation/acetate symporter, likely altering the strain’s capacity for acetate and small aliphatic acid metabolisms (89, 90); multiple mutations were detected in transcriptional and stress response genes of Hd, including *rpoC*, *rpoD*, and *clpX*, which are known to regulate oxidative stress tolerance, general stress responses, and virulence factor expression (91–97). Therefore, these findings suggest that adaptive mutations are an important driver of microbial evolution at the gene level, which is suggestive of the Red Queen Hypothesis (15).

Despite the accumulation of diverse mutations across populations, we only identified a single newly acquired but non-fixed mutation directly associated with N cycling genes, indicating that denitrification pathways remain genetically and functionally conserved, which is important for sustaining community stability. Thus, this study provides a new insight that microbial interaction-driven adaptive mutations may largely occur peripherally but functionally relevant genes rather than core functional genes. Interestingly, we also found the parallel emergence of functionally similar mutations across the replicates of SDC6, which underscores the predictability of adaptive trajectories and highlights how synthetic microbial communities can be used as a powerful platform to uncover general principles of eco-evolutionary dynamics (78, 81). Taken together, our results demonstrate that mutations act as a key driver of functional adaptation and evolution in complex communities, while ecological interactions shape the direction of fixed mutations, thus improving community functions.

**Fig 7:**
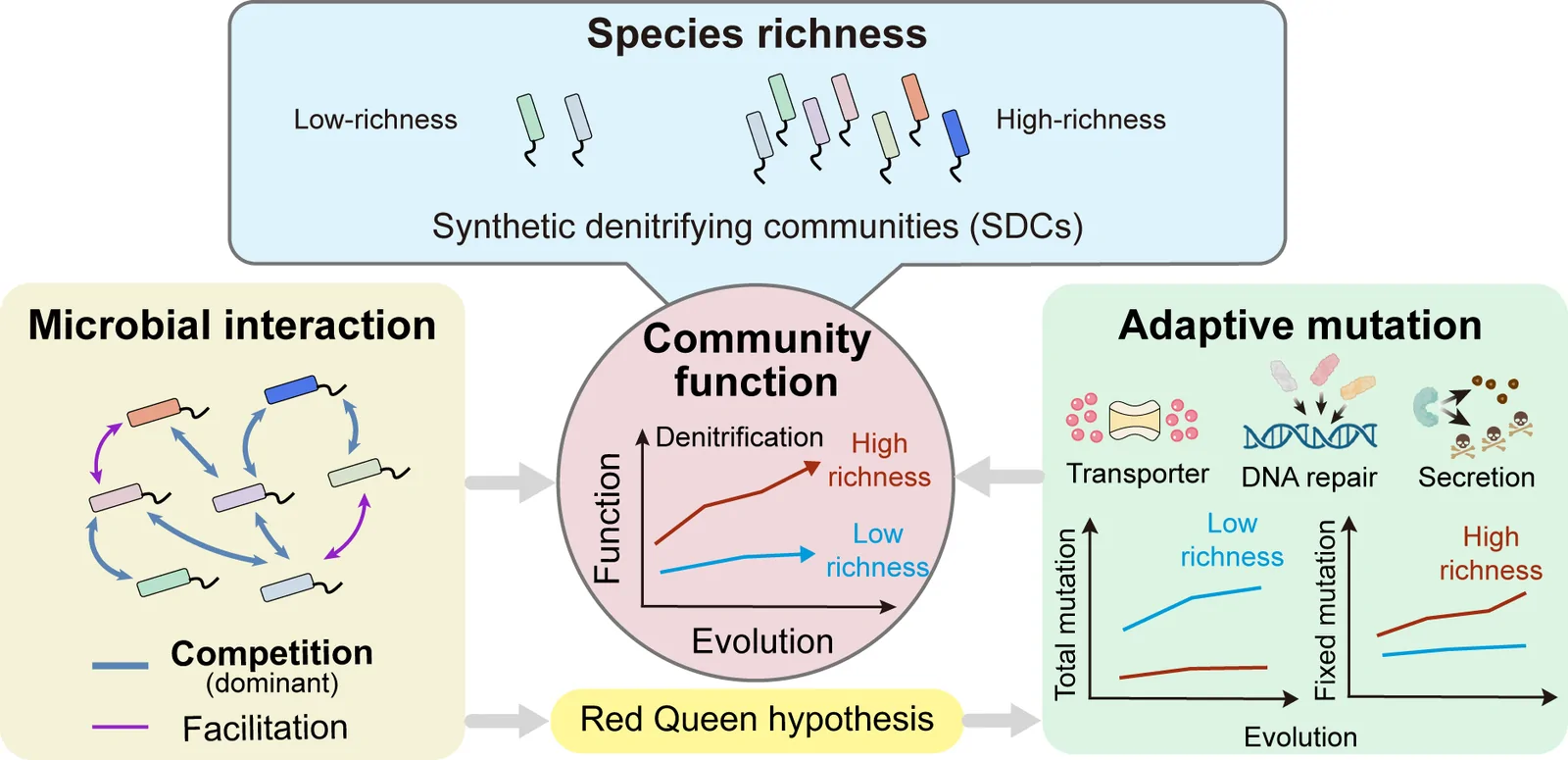
Conceptual model of the potential mechanisms for the relationship between species richness, microbial interaction, adaptive mutation, and community function during long-term experimental evolution.

In summary, this study aims to understand how species richness, microbial interactions, and adaptive mutations jointly shape community functions using synthetic microbial ecology and experimental evolution approaches (Fig. 7). We found that microbial competition in high-richness SDCs promoted the accumulation of beneficial mutations, especially for the genes involved in transport, DNA repair, and secretion systems through Red Queen-type dynamics, ultimately enhancing community functions. These findings provide novel insights into our understanding of how microbial interactions and adaptive mutations shape community functioning, highlighting the importance of integrating ecological and evolutionary perspectives for richness-interaction-evolution studies. This study has important implications for developing evolutionarily robust synthetic microbial communities to remove excessive N and reduce greenhouse gas emissions in various ecosystems toward microbiome engineering. Future research should test the Red Queen hypothesis and determine the functional roles of specific mutations through competitive fitness assays, gene editing, isotope labeling, and multi-omics technologies, thereby quantifying their contributions to denitrification.

## MATERIALS AND METHODS

### Bacterial strains and growth conditions

Six bacterial strains with complete genome information were selected for this study, including *Paracoccus denitrificans* (Pd; ATCC 19367), *Desulfovibrio vulgaris* Hildenborough (DvH; DSM 644), *Brucella anthropi* (Ba; ATCC 49188), *Providencia rustigianii* (Pr; ATCC 33673), *Hyphomicrobium denitrificans* (Hd; ATCC 51888), and *Thauera aromatica* K172 (Ta; DSM 6984) (Table S6). Notably, DvH is not a canonical denitrifier and was intentionally included as a functionally distinct nitrogen-transforming strain. DvH carries the nrfA/H nitrite-reduction system, providing the potential to compete with denitrifying community members for nitrite as a shared nitrogen intermediate. Each strain was cultured in the M13 medium (Table S1) at 32°C under static and anaerobic conditions. Bacterial strains and culture conditions were selected based on our previous study with SDCs (23), showing that these species consistently coexisted in the community during long-term experimental evolution.

### Construction of SDCs and evolution experiment

We constructed SDCs with the species richness levels of one (SDC1), two (SDC2), and six species (SDC6). This experimental design was based on previous plant or microbial diversity–ecosystem functioning studies in which different species compositions were treated as replicates at each richness level (7, 53, 98). In this study, we focused on six bacterial strains, as only these six species could coexist during long-term experimental evolution, although the original pool was 10 species (23). For SDC1, each of the six species was cultivated individually, and these mono-cultures were treated as six independent replicates. The three randomly selected two-species SDCs were DvH–Pd, Pr–Ba, and Ta–Hd, and each pair was treated as one replicate with three replicates at the two-species richness level. For SDC6, all six species were grown together with three biological replicates. To ensure an equal total initial cell number in each SDC, we used a hemocytometer to quantify mono-cultures at the exponential phase and diluted them to the same cell concentration (7.05 × 10^6^ cells/mL). Then, we mixed the strains in equal proportions corresponding to the composition of two-species or six-species SDCs and added 0.1 mL of the mixed bacterial solution to 9.9 mL of fresh anaerobic M13 medium. These SDCs reached the exponential phase after 5.5 days, so we transferred 1% of each SDC into fresh medium every 5.5 days. This evolution experiment was carried out for a total of 28 serial transfers, approximately 6 months.

We measured the community productivity, nitrate consumption rates, nitrite production rates, and absolute abundances of strains, and extracted community DNA in the start communities (before the 1st transfer, *T*_0_), as well as at the 7th, 14th, 21st, and 28th transfers, as described in the Supplemental material.

### DNA extraction and genome/metagenome re-sequencing

We collected 1–9 mL of SDC cultures (depending on biomass concentration) and centrifuged them at 10,000 × *g* for 1 min, discarded the supernatant, and washed them repeatedly with 1 mL of sterile phosphate buffered saline (PBS) solution three times. Genomic DNA was extracted using a pure bacterial DNA extraction kit (Guangdong Meige Gene Technology Co., Ltd.). The DNA concentration and quality (A260/A230 and A260/A280) were detected using a NanoDrop ND-2000 spectrophotometer (Thermo Fisher Scientific, USA). All SDC samples were subjected to shotgun metagenome sequencing, as described in the Supplemental material.

### Quantification of microbial interactions

To assess microbial interactions, we compared absolute abundance changes in strains in multi-species communities (SDC2 and SDC6) relative to mono-cultures (SDC1), expressed as log_10_-transformed ratios. The OIF in SDCs was calculated as:


$$OIF=\frac{1}{n}\sum log\left(\frac{{CP}_{i}}{{MP}_{i}}\right),$$

where MP_*i*_ represents the mono-culture absolute abundance of strains *i*, CP_*i*_ is the absolute abundance in the co-culture, and *n* means species richness.

Communities were defined as facilitative when OIF > 0, antagonistic when OIF < 0, and neutral if OIF = 0, as described previously (59, 99). In SDC2, we determined the directionality of interaction as facilitative if species *j* had a positive effect on *i* [log (CP_*i*_/MP_*i*_) >0], antagonistic if log (CP_*i*_/MP_*i*_) < 0, and neutral if log (CP_*i*_/MP_*i*_) = 0. In SDC6, Spearman correlation analysis was applied to infer interactions between species.

### Mutation calling and annotation

The reference genomes of six bacterial strains were downloaded from National Center for Biotechnology Information (NCBI) Genome or ATCC Genome Portal (Table S6). The indexes were created, and the clean reads were mapped to the reference genome using Bowtie2 (version 2.4.5) with the default settings (100). Mutations were called using bcftools mpileup and bcftools call in BCFtools (version 1.18). We provide a detailed description of these steps in the Supplemental material. The AF of each variant was inferred using the DP4 field in the output variant call format (VCF) file, which provides the number of high-quality reference and alternative base calls on both strands. The formula used was as follows:


$$AF=\;\frac{alt\_fwd+alt\_rev}{ref\_fwd+ref\_rev+alt\_fwd+alt\_rev}.$$

We annotated and predicted the effects of the mutations using SnpEff (version 4.3) (101). The mutation types annotated in this study include non-synonymous mutation, frameshift mutation, synonymous mutation, and intergenic mutation. The mutation rate (μ) of individual populations or SDCs was calculated using the following formula:


$$\mu =\frac{M}{L},$$

where *M* is the total number of mutations observed in an individual population or SDC and *L* is the total number of bases (in millions [Mbp]) in an SDC obtained with SeqKit2 (102).

Total mutations included all variants detected relative to the corresponding reference genome, including variants present in the start communities. Newly acquired mutations were identified after excluding variants already detected in the start communities. Fixed mutations were defined as newly acquired mutations with AF ≥0.90 (103). Functionally annotated fixed mutations refer specifically to fixed mutations located in genes with an assignable functional annotation. We only selected functionally annotated fixed non-synonymous and frameshift mutations detected at the last two transfers for subsequent protein structure predictions.

### Protein structure prediction, fitness prediction, and molecular docking

As non-synonymous and frameshift mutations may change protein structures, we used AlphaFold2 to predict the protein structure (104). We also employed Evo 2 to evaluate the potential fitness impacts of fixed mutations on microbial strains (105). Two representative fixed mutations (DVU_RS15435 and OANT_RS07400) were selected for molecular docking using AutoDock Tools (version 1.5.7) and AutoDock 4.2.6 (The Scripps Research Institute, USA). We provide a detailed description of these analyses in the Supplemental material.

### Partial least squares path modeling

To investigate the general patterns underlying the relationships between species richness, microbial interaction, fixed mutation, and community function, we first constructed a PLS-PM. Four latent variables were defined: species richness, microbial interaction, fixed mutation, and community function. Microbial interaction is defined as the absolute log_10_ fold change in the abundance of each strain in SDC6 or SDC2 relative to SDC1; community function was represented by community productivity, nitrate consumption rate, and nitrite production rate. We tested if (i) species richness, interaction effect, and fixed mutation collectively would influence community function; (ii) species richness and interaction effect would determine the fixed mutation; and (iii) species richness would affect interaction effect. The models were implemented in R (R Foundation for Statistical Computing, Vienna, Austria) using the *plspm* and *dplyr* packages. Model quality was evaluated using the goodness-of-fit index.

### Statistical analysis

In this study, most statistical analyses were conducted using SPSS 22.0 (SPSS Inc., Chicago, IL, USA) and Origin 2021 (OriginLab Corporation, Northampton, MA, USA), while PLS-PM was performed in R. Two-way ANOVA was performed to evaluate the effects of species richness and evolution time, as well as their interaction, on microbial community productivity, nitrate consumption rate, nitrite production rate, and community mutation rate. The effects of species richness, evolution time, and their interaction on mutation rates were also tested by two-way ANOVA. One-way ANOVA was used to compare mutation rates among the six species, community-level mutation rates across transfer time points in SDC6, and community-level OIF values among treatments. Spearman correlation analysis was conducted to estimate correlations between mutation rate and productivity, nitrate consumption rate, and nitrite production rate. Paired *t*-test and one-way ANOVA were performed to compare absolute abundances of each species in SDC1 with those in its paired two- and six-species SDCs, respectively (11, 58). Duncan’s multiple range test was used to assess significant differences among group means. In all tests, *P* < 0.05 was considered statistically significant.

## Data Availability

Metagenomic data for 60 synthetic denitrifying community samples are available in the NCBI Sequence Read Archive under BioProject accession PRJNA1190945.

## SUPPLEMENTAL MATERIAL

Supplemental Material mbio.02360-26-s0001.pdf Supplemental methods and Fig. S1-S9.

Supplemental Tables mbio.02360-26-s0002.xlsx Tables S1-S7.

ASM does not own the copyrights to Supplemental Material that may be linked to, or accessed through, an article. The authors have granted ASM a non-exclusive, world-wide license to publish the Supplemental Material files. Please contact the corresponding author directly for reuse.
