# Supplementary material for: Competition-dominated microbial interactions and adaptive mutations enhance synthetic denitrifying community functions
Source: mBio. 2026 Oct 21;17(11):e02360-26. doi: 10.1128/mbio.02360-26 (PMC13753840; doi:10.1128/mbio.02360-26)
Supplement: Supplementary file 1 [file mbio.02360-26-s0001.pdf]

Supplementary Materials for

Competition-dominated microbial interactions and adaptive mutations enhance synthetic denitrifying community functions

Zhenrui Zhao[a], Ruiwen Hu[b]#, Min Zhou[a], Xiaotong Guan[a], Huaxia Huang[a], Lang Ding[a], Lu Qian[a], Yuzhen Ming[a], Mingyang Niu[a], Kun Wu[a], Xiaoli Yu[a], Qingyun Yan[a], Longfei Shu[a], Qiang He[c], Zhili He[a]#

[a]School of Marine Sciences, Marine Synthetic Ecology Research Center, Southern Marine Science and Engineering Guangdong Laboratory (Zhuhai), the China-ASEAN Belt and Road Joint Laboratory on Mariculture Technology, School of Environmental Science and Engineering, Sun Yat-sen University, Zhuhai 519082, China

[b]Environmental Genomics and Systems Biology Division, Lawrence Berkeley National Laboratory, Berkeley, CA, 94720, USA

[c]Department of Civil and Environmental Engineering, University of Tennessee, Knoxville, TN, 37996, USA

#Address correspondence to Ruiwen Hu (RuiwenHu@lbl.gov) and Zhili He (hezhili@sml-zhuhai.cn)

Contents

Supplementary Materials and Methods ……………………………………………..…Pages 4-8

Determination of community productivity, nitrate consumption rates, nitrite production rates and absolute abundances

Genome/metagenome re-sequencing and quality control of sequencing data

Mutation calling and annotation

Protein structure prediction, fitness prediction and molecular docking

References

Supplementary Figures ……………………………………………………………Pages 9-17

Fig. S1. Absolute abundance changes of six strains in SDC1 (a), SDC2 (b) and SDC6 (c) during the experimental evolution.

Fig. S2. Changes of total mutation rates of Pd (a), DvH (b), Ba (c), Pr (d), Ta (e) and Hd (f) strains in different SDCs during the experimental evolution.

Fig. S3. Changes of newly acquired mutation rates of Pd (a), DvH (b), Ba (c), Pr (d), Ta (e) and Hd (f) strains in different SDCs during the experimental evolution.

Fig. S4. Changes of fixed mutation rates of Pd (a), DvH (b), Ba (c), Pr (d), Ta (e) and Hd (f) strains in different SDCs during the experimental evolution.

Fig. S5. Comparison of total, newly acquired, and fixed mutation rates among the six bacterial strains.

Fig. S6. Spearman correlation between three types of mutation rates (total, newly acquired, fixed) and three community functional traits (productivity, nitrate consumption rate, nitrite production rate) during the experimental evolution.

Fig. S7. Dynamic changes in mutation allele frequency for DVU_RS15435, EO213_RS17915, OANT_RS07400, Tharo_RS10060 and HDEN_RS09645 during the experimental evolution.

Fig. S8. Protein structure prediction before (left) and after (right) mutations.

Fig. S9. Protein structure prediction and comparison of ATP binding energies (kcal/mol) before and after mutation in DVU_RS15435 based on molecular docking analysis.

Supplementary Materials and Methods

Determination of community productivity, nitrate consumption rates, nitrite production rates and absolute abundances

We used a V-1000 spectrophotometer (AOE Instruments, China) to monitor optical density at 600 nm (OD600) values of all SDCs every 3 h and recorded the maximum OD600 values as the community productivity. In addition, we collected 1 mL of cultures at the beginning (t_1) and end (t_2) of the log phase to determine the nitrate concentration ($C_{nitrate}$) and nitrite concentration ($C_{nitrite}$) by an ion chromatograph (Dionex ICS-1100, Thermo Scientific, USA), as described by Zhou et al.(1). The nitrate consumption and nitrite production rates were calculated by $(C_{nitrate}(t_1) - C_{nitrate}(t_2))/(t_1 - t_2)$ and $(C_{nitrite}(t_2) - C_{nitrite}(t_1))/(t_1 - t_2)$, respectively.

We used quantitative PCR (qPCR) to quantify the absolute abundance of each strain of SDCs. To generate the standard curve, qPCR was performed with various concentrations of plasmids containing the target gene fragments. As previously described (2, 3), these fragments were amplified by PCR using the corresponding genomic DNA and primers (Table S7). The purified PCR products were ligated into plasmid vectors using the pEASY®-T1 Simple Cloning Kit (TransGen Biotech, Beijing, China). Transformed *Escherichia coli* competent cells containing the target gene fragment were identified through Blue-White Screening. Selected monoclonal colonies were amplified, and plasmids were extracted using the DiaSpin column plasmid DNA extraction kit (Sangon Biotechnology, China). These standard samples were then sequenced for verification and the concentration was determined with a Qubit® 3.0 Fluorometer. The qPCR reaction system was 12 μL, consisting of 6 μL of iTaq™ Universal SYBR Green Supermix (BIO-RAD, USA), 0.5 μL each of forward and reverse primers, 2 μL of sample, and 3 μL of deionized water. The qPCR protocol included an initial denaturation step at 95°C for 4 minutes,

followed by 45 cycles of amplification, each cycle comprising denaturation at 95°C for 30 seconds, annealing at 62°C for 30 seconds, and extension at 72°C for 15 seconds. The dissolution curve analysis ranged from 65°C to 95°C, with increments of 0.2°C per second.

Genome/metagenome re-sequencing and quality control of sequencing data

All SDC samples were subjected to shotgun metagenome sequencing. DNA sequencing libraries were prepared using the NEBNext® UltraTM DNA Library Prep Kit for Illumina (NEB, USA) following the manufacturer's protocol. The DNA concentrations of the prepared libraries were quantified using a Qubit HS DNA assay, and the library insert sizes were assessed using an Agilent 2100 Bioanalyzer with a High Sensitivity DNA chip. Cluster generation was performed using a cBot Cluster Generation System, followed by paired-end sequencing (PE150) on an Illumina platform at Guangdong Magigene Biotechnology Co., Ltd. The sequencing depth varied across samples: SDC1 at 1Gb, SDC2 at 20Gb, and SDC6 at 50Gb to ensure adequate coverage (250X ~ 2,000X) for all constituent strains. After adapter trimming and quality filtering, reads containing more than 10% unknown bases ('N') or more than 50% low-quality bases (quality score⩽5) were removed to ensure high-quality data for downstream analysis.

Mutation calling and annotation

To identify mutations in the community level and individual population level, we used BBDuk (k = 28, mink = 12, trimq = 20, minlength = 70) (https://jgi.doe.gov/data-and-tools/software-tools/bbtools/bb-tools-user-guide/bbduk-guide/) for quality control of the raw data and FastQC (version 0.12.1, http://www.bioinformatics.babraham.ac.uk/projects/fastqc/) to assess the quality of the clean data. The reference genomes of six bacterial strains were downloaded from NCBI Genome or ATCC Genome Portal (Table S6). The indexes were created

and the clean reads were mapped to the reference genome using Bowtie2 (version 2.4.5) with the default settings (4). Then we converted the obtained SAM file into a sorted BAM file by SAMtools (version 1.15) (5). Mutations were called using bcftools mpileup and bcftools call command with parameters "-c -v" in BCFtools (version 1.18), which enable consensus variant calling and variant-only output (6). To ensure high confidence of the mutation, we used bcftools filter command to filter out unreliable mutations with coverage depth (DP) less than 10 and phred-scaled quality score (QUAL) less than 20 (corresponding to a base call accuracy of over 99%). These filtering thresholds help eliminate sequencing artifacts and low-confidence polymorphisms.

Protein structure prediction, fitness prediction and molecular docking

As non-synonymous and frameshift mutations may change protein structures, we used AlphaFold2 to predict the protein structure (7). The protein structure before and after gene mutations was compared using the PyMOL software (Schrödinger, LLC) (8). We used the default parameters for alignment (cycles = 5; cutoff = 2.0) and RMSD (root-mean-square deviation) to assess the dissimilarity of protein structures between before and after mutations (lower value for more similarity). We also employed Evo 2 (9) upgraded from Evo 1 (10) to evaluate the potential fitness impacts of fixed mutations on microbial strains. Evo 2 is a deep learning framework integrating DNA, RNA or protein mutation embeddings with organismal fitness prediction. In the output file, "impact_score" represents the predicted effect of mutations on host fitness with a high value as a high adaptive potential (9).

Two representative fixed mutations (DVU_RS15435 and OANT_RS07400) were selected for molecular docking using AutoDock Tools (version 1.5.7) and AutoDock 4.2.6 (The Scripps Research Institute, USA). Ligand structures, such as ATP and urocanate, were obtained from the

Protein Data Bank (https://www.rcsb.org) in CIF format. For docking preparation, the target protein structure was first processed by removing all water molecules and adding polar hydrogens. The processed protein was then saved in PDBQT format and defined as the receptor for subsequent docking procedures. Ligand structures were similarly prepared by adding all hydrogens, assigning Kollman charges, and detecting rotatable bonds. Rotatable torsions were automatically identified, and the flexible bonds were manually confirmed. The ligand was subsequently saved in PDBQT format and defined as the ligand in the docking process. A grid box was constructed to define the binding region of the receptor. Docking simulations were carried out using the Lamarckian Genetic Algorithm (LGA). The docking parameters included a population size of 150, a maximum of 2,500,000 energy evaluations, and up to 27,000 generations. The number of runs ranged from 10 to 100, and the mutation and crossover rates were set to 0.02 and 0.8, respectively. A two-point crossover operator was employed for all runs. The primary scoring criterion for binding affinity was the binding free energy (G), with additional parameters including the inhibition constant (K_i), hydrogen bond formation, and the number and identity of interacting amino acid residues. Among all conformations generated, the one with the most negative G and the lowest K_i value was selected as the optimal binding pose. PyMOL was used for visualization and structural analysis of docked complexes.

References

1. Zhou M, Guan X, Deng T, Hu R, Qian L, Yang X, Wu B, Li J, He Q, Shu L, Yan Q, He Z. 2023. Synthetic phylogenetically diverse communities promote denitrification and stability. Environ Res 231:116184.
2. Tu Q, He Z, Deng Y, Zhou J. 2013. Strain/species-specific probe design for microbial identification microarrays. Appl Environ Microbiol 79:5085-5088.

3. Tu Q, He Z, Zhou J. 2014. Strain/species identification in metagenomes using genome-specific markers. Nucleic Acids Res 42:e67.
4. Langmead B, Salzberg SL. 2012. Fast gapped-read alignment with Bowtie 2. Nat Methods 9:357-359.
5. Li H, Handsaker B, Wysoker A, Fennell T, Ruan J, Homer N, Marth G, Abecasis G, Durbin R, Genome Project Data Processing S. 2009. The Sequence Alignment/Map format and SAMtools. Bioinformatics 25:2078-9.
6. Narasimhan V, Danecek P, Scally A, Xue Y, Tyler-Smith C, Durbin R. 2016. BCFtools/RoH: a hidden Markov model approach for detecting autozygosity from next-generation sequencing data. Bioinformatics 32:1749-1751.
7. Jumper J, Evans R, Pritzel A, Green T, Figurnov M, Ronneberger O, Tunyasuvunakool K, Bates R, Zidek A, Potapenko A, Bridgland A, Meyer C, Kohl SAA, Ballard AJ, Cowie A, Romera-Paredes B, Nikolov S, Jain R, Adler J, Back T, Petersen S, Reiman D, Clancy E, Zielinski M, Steinegger M, Pacholska M, Berghammer T, Bodenstein S, Silver D, Vinyals O, Senior AW, Kavukcuoglu K, Kohli P, Hassabis D. 2021. Highly accurate protein structure prediction with AlphaFold. Nature 596:583-589.
8. Schrodinger, LLC. 2015. The PyMOL Molecular Graphics System, Version 3.1.
9. Brixi G, Durrant MG, Ku J, Poli M, Brockman G, Chang D, Gonzalez GA, King SH, Li DB, Merchant AT, Naghipourfar M, Nguyen E, Ricci-Tam C, Romero DW, Sun G, Taghibakshi A, Vorontsov A, Yang B, Deng M, Gorton L, Nguyen N, Wang NK, Adams E, Baccus SA, Dillmann S, Ermon S, Guo D, Ilango R, Janik K, Lu AX, Mehta R, Mofrad MRK, Ng MY, Pannu J, Ré C, Schmok JC, John JS, Sullivan J, Zhu K, Zynda G, Balsam D, Collison P, Costa AB, Hernandez-Boussard T, Ho E, Liu M-Y, McGrath T, Powell K, Burke DP, Goodarzi H, et al. 2025. Genome modeling and design across all domains of life with Evo 2. bioRxiv doi:10.1101/2025.02.18.638918:2025.02.18.638918.
10. Nguyen E, Poli M, Durrant MG, Kang B, Katrekar D, Li DB, Bartie LJ, Thomas AW, King SH, Brixi G, Sullivan J, Ng MY, Lewis A, Lou A, Ermon S, Baccus SA, Hernandez-Boussard T, Re C, Hsu PD, Hie BL. 2024. Sequence modeling and design from molecular to genome scale with Evo. Science 386:eado9336.

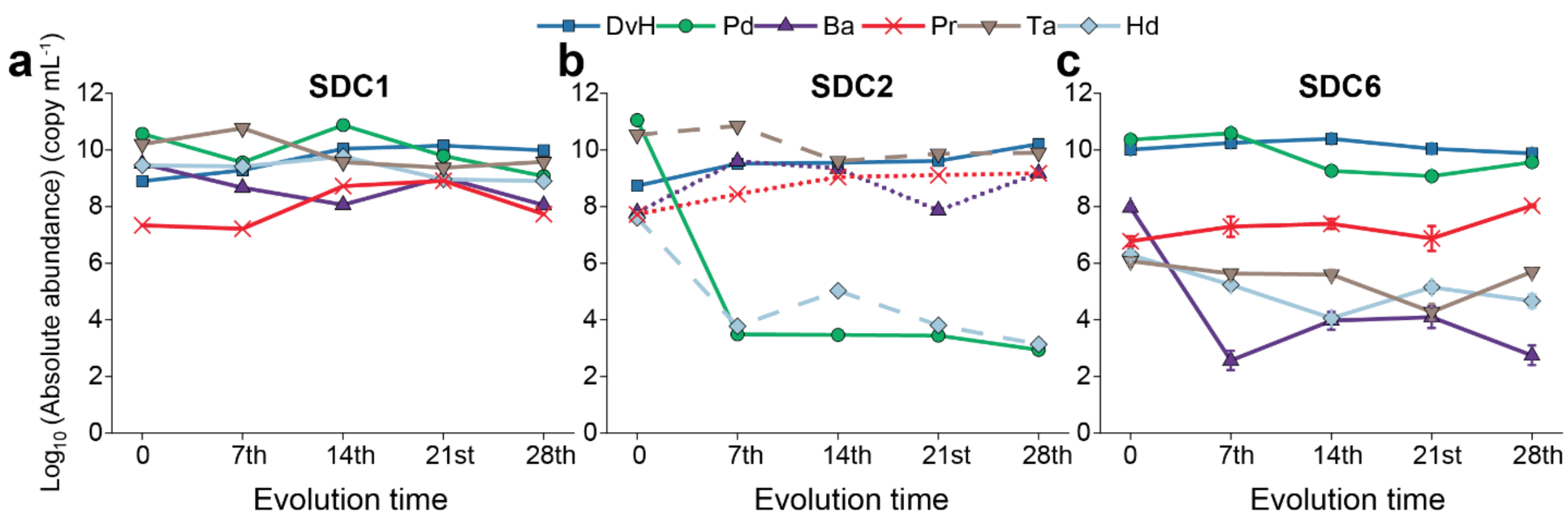

Fig. S1. Absolute abundance changes of six strains in SDC1 (**a**), SDC2 (**b**) and SDC6 (**c**) during experimental evolution. Data in SDC6 are presented as mean ± standard error. The same line in SDC2 represents two strains co-evolved in the same community.

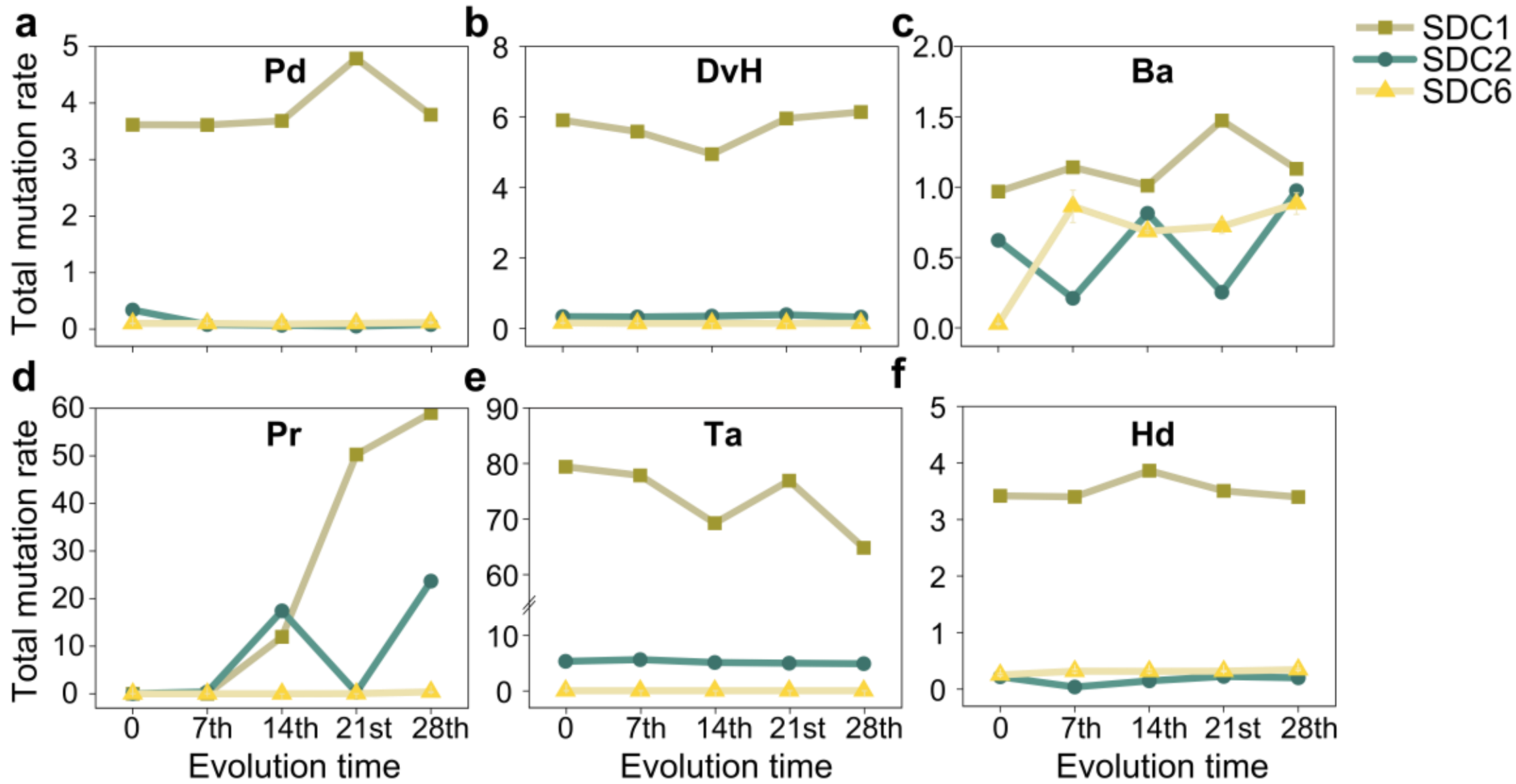

Fig. S2. Changes of total mutation rates of Pd (**a**), DvH (**b**), Ba (**c**), Pr (**d**), Ta (**e**) and Hd (**f**) strains in different SDCs during the experimental evolution.

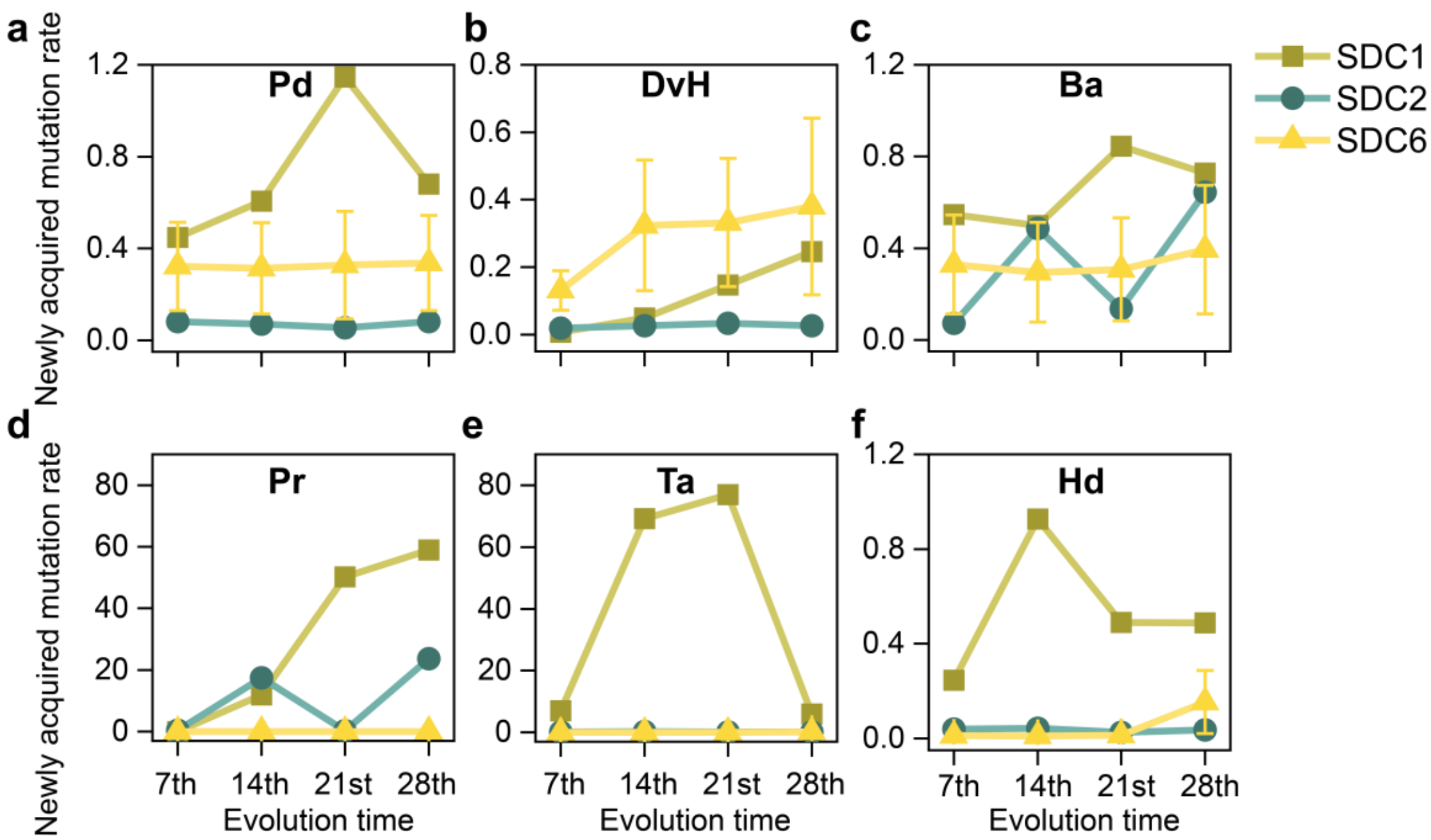

Fig. S3. Changes of newly acquired mutation rates of Pd (a), DvH (b), Ba (c), Pr (d), Ta (e) and Hd (f) strains in different SDCs during the experimental evolution.

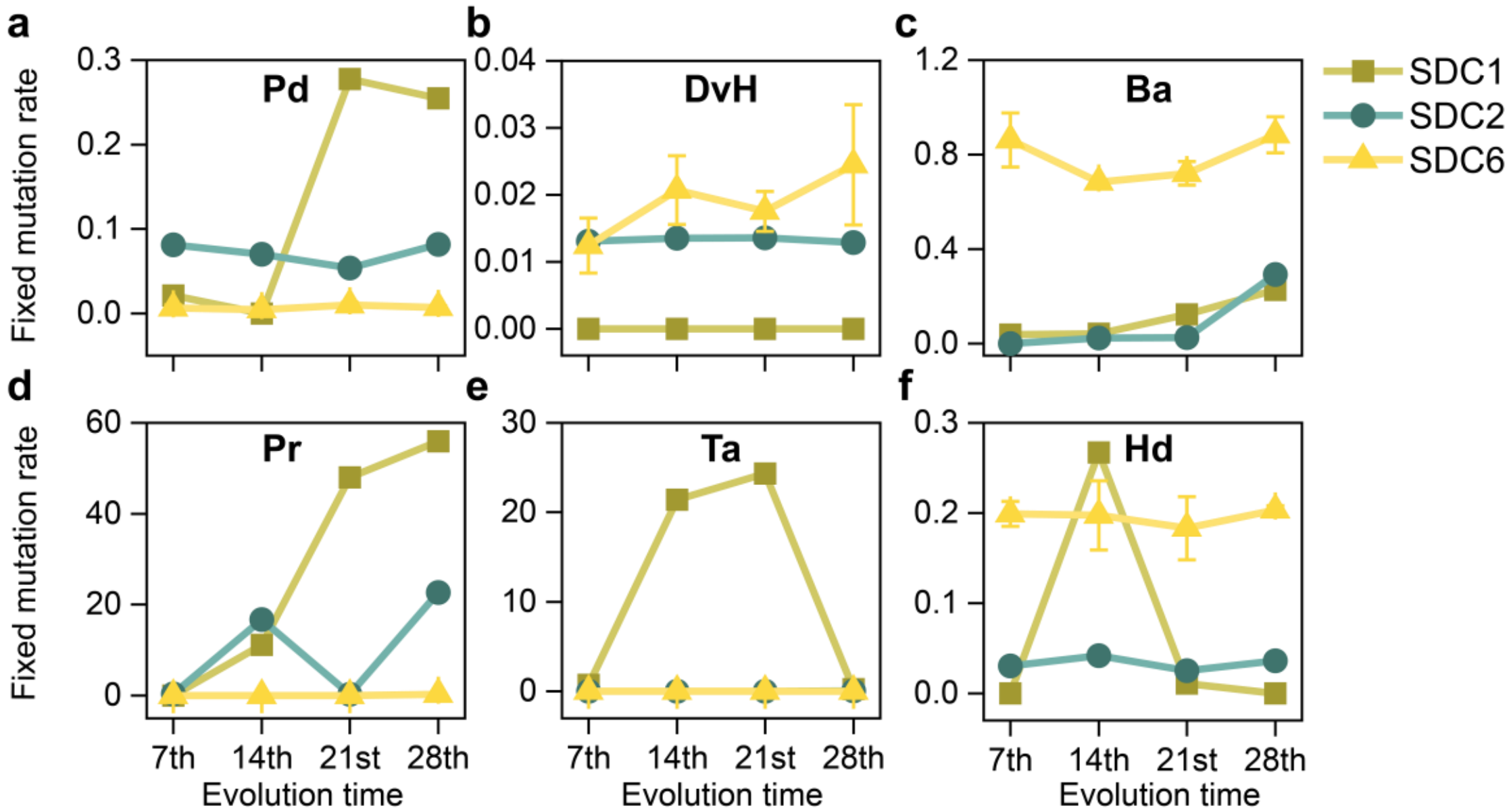

Fig. S4. Changes of fixed mutation rates of Pd (**a**), DvH (**b**), Ba (**c**), Pr (**d**), Ta (**e**) and Hd (**f**) strains in different SDCs during the experimental evolution.

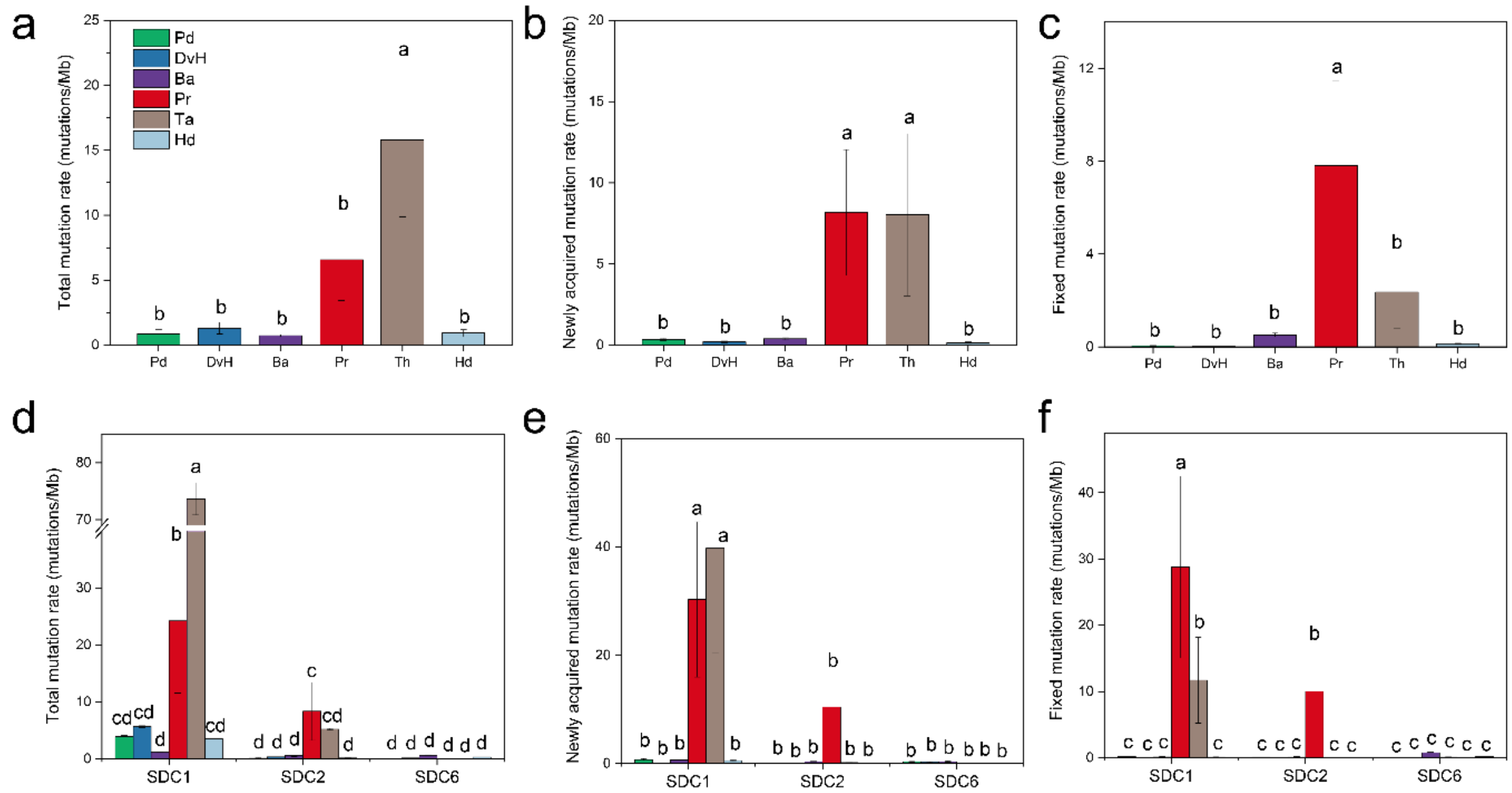

Fig. S5. Comparison of total, newly acquired, and fixed mutation rates among the six bacterial strains. (a-c) Overall total (a), newly acquired (b), and fixed (c) mutation rates of the six species (Pd, DvH, Ba, Pr, Th, and Hd). (d-f) The total (d), newly acquired (e), and fixed (f) mutation rates of the six species in SDC1, SDC2, and SDC6. Data are presented as mean ± SE. Different lowercase letters indicate significant differences ($P < 0.05$).

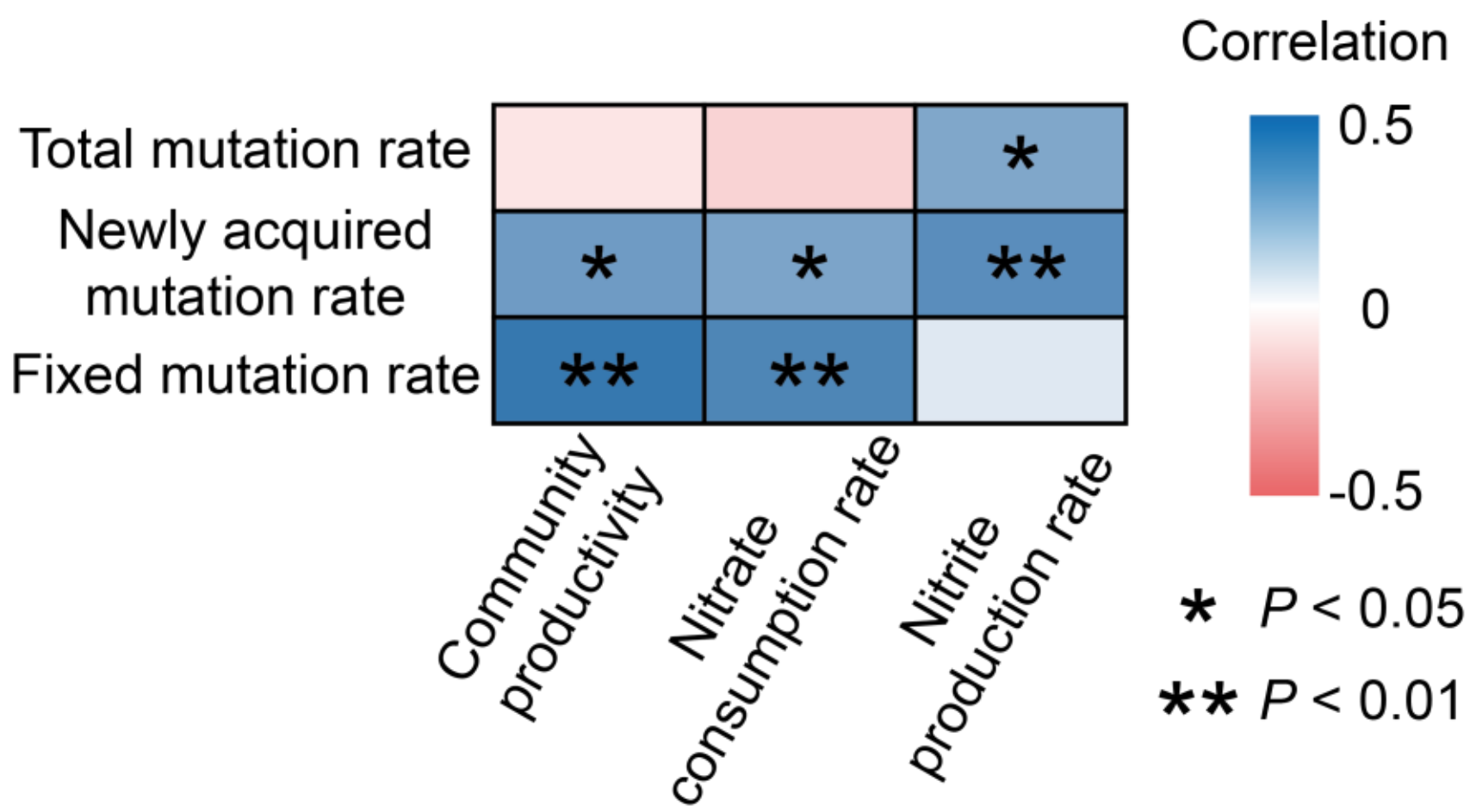

Fig. S6. Spearman correlation between three types of mutation rates (total, newly acquired, fixed) and three community functional traits (productivity, nitrate consumption rate, nitrite production rate) during the experimental evolution.

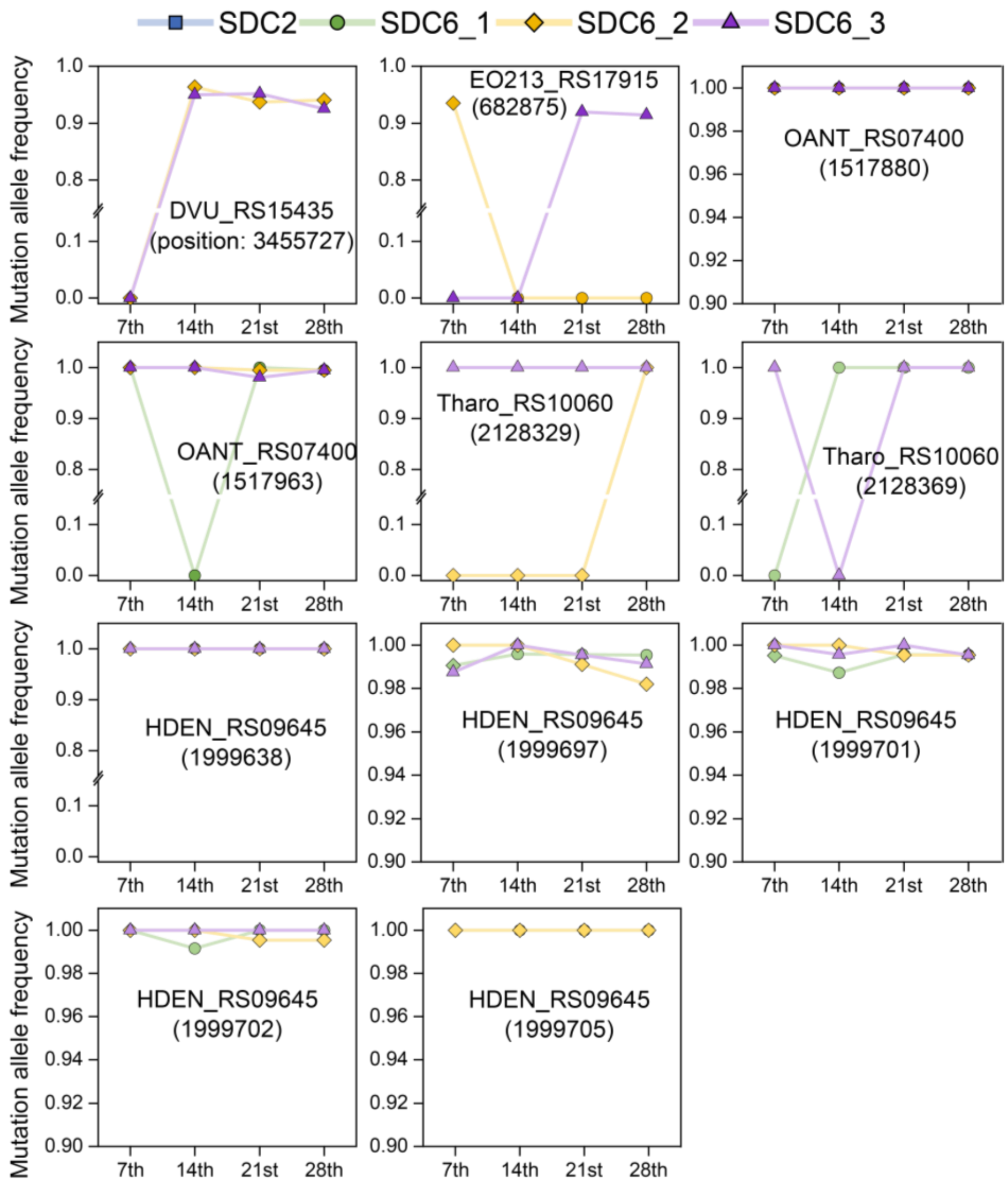

Fig. S7. Dynamic changes of mutation allele frequency for DVU_RS15435, EO213_RS17915, OANT_RS07400, Tharo_RS10060 and HDEN_RS09645 during the experimental evolution.

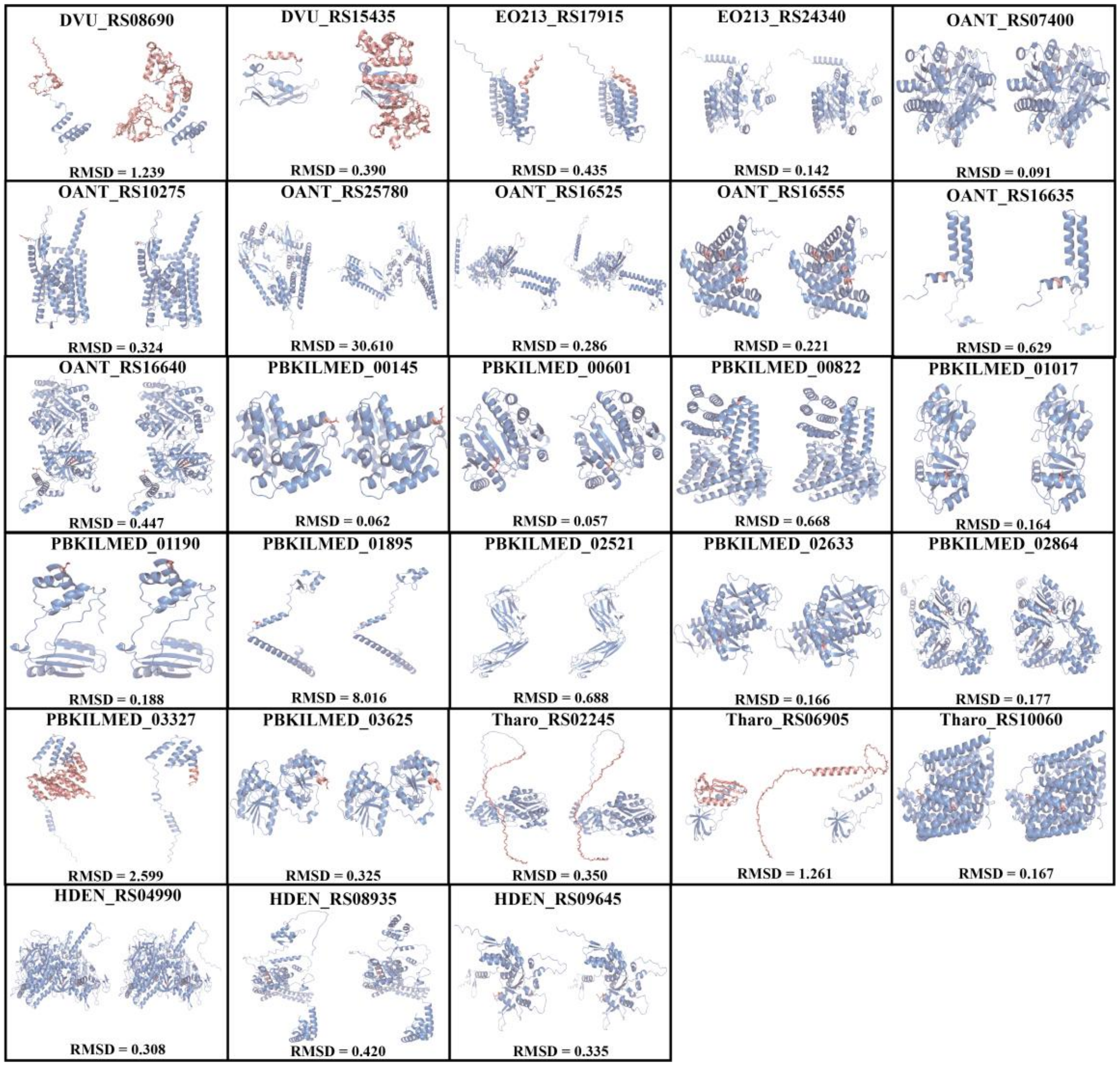

Fig. S8. Protein structure prediction before (left) and after (right) mutations. Red protein structure represents the mutation position. RMSD (root-mean-square deviation) value is used to assess the dissimilarity of protein structures between before and after mutations (lower value for more similarity).

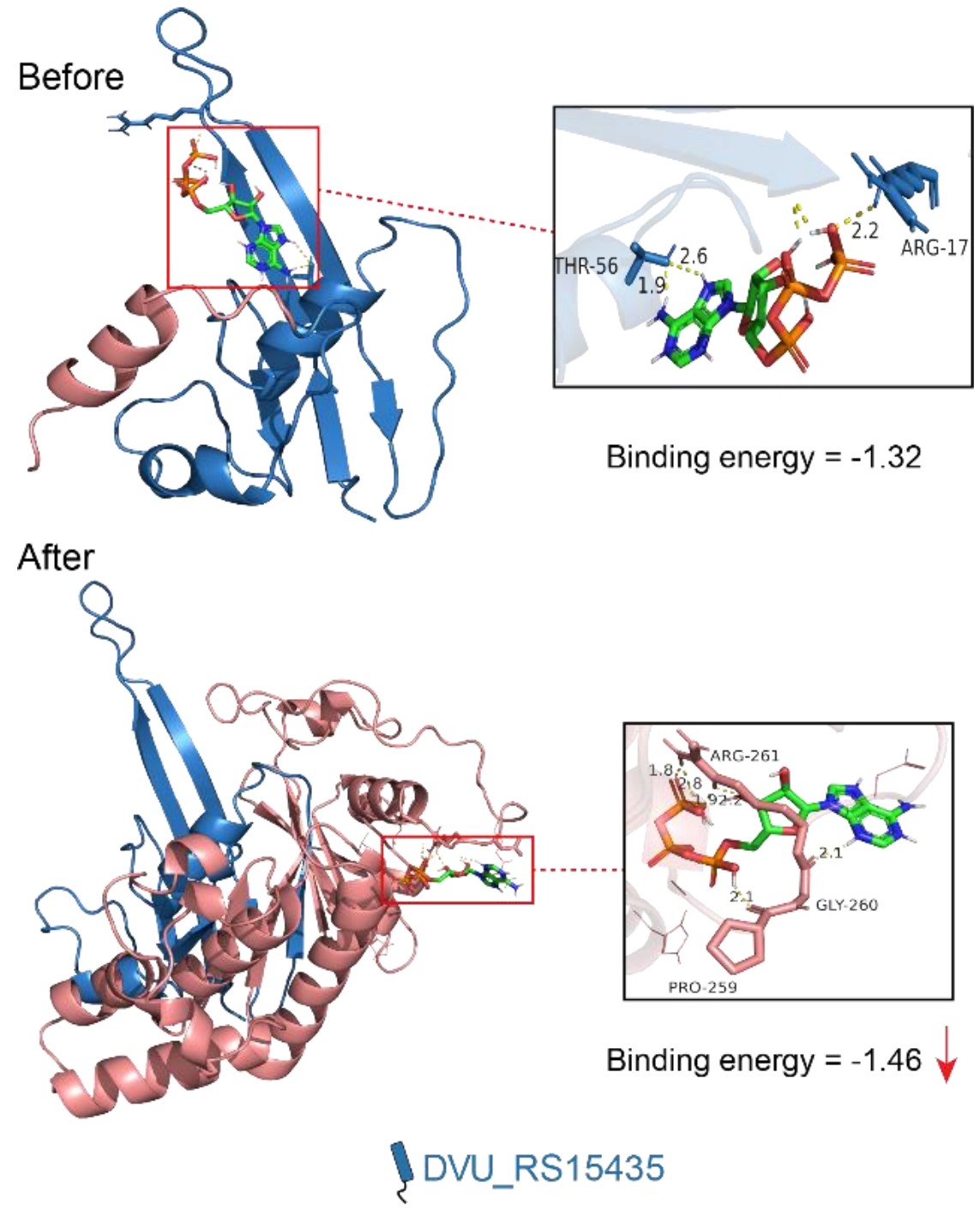

Fig. S9. Protein structure prediction and comparison of ATP binding energies (kcal/mol) before and after mutation in DVU_RS15435 based on molecular docking analysis.
